# The Small Molecule DAM Inhibitor, Pyrimidinedione, Disrupts *Streptococcus pneumoniae* Biofilm Growth *In Vitro*

**DOI:** 10.1371/journal.pone.0139238

**Published:** 2015-10-02

**Authors:** Mukesh Kumar Yadav, Yoon Young Go, Sung-Won Chae, Jae-Jun Song

**Affiliations:** 1 Department of Otorhinolaryngology-Head and Neck Surgery, Korea University College of Medicine, Seoul, South Korea; 2 Institute for Medical Device Clinical Trials, Korea University College of Medicine, Seoul, South Korea; Centers for Disease Control & Prevention, UNITED STATES

## Abstract

*Streptococcus pneumoniae* persist in the human nasopharynx within organized biofilms. However, expansion to other tissues may cause severe infections such as pneumonia, otitis media, bacteremia, and meningitis, especially in children and the elderly. Bacteria within biofilms possess increased tolerance to antibiotics and are able to resist host defense systems. Bacteria within biofilms exhibit different physiology, metabolism, and gene expression profiles than planktonic cells. These differences underscore the need to identify alternative therapeutic targets and novel antimicrobial compounds that are effective against pneumococcal biofilms. In bacteria, DNA adenine methyltransferase (Dam) alters pathogenic gene expression and catalyzes the methylation of adenine in the DNA duplex and of macromolecules during the activated methyl cycle (AMC). In pneumococci, AMC is involved in the biosynthesis of quorum sensing molecules that regulate competence and biofilm formation. In this study, we examine the effect of a small molecule Dam inhibitor, pyrimidinedione, on *Streptococcus pneumoniae* biofilm formation and evaluate the changes in global gene expression within biofilms via microarray analysis. The effects of pyrimidinedione on *in vitro* biofilms were studied using a static microtiter plate assay, and the architecture of the biofilms was viewed using confocal and scanning electron microscopy. The cytotoxicity of pyrimidinedione was tested on a human middle ear epithelium cell line by CCK-8. *In situ* oligonucleotide microarray was used to compare the global gene expression of *Streptococcus pneumoniae* D39 within biofilms grown in the presence and absence of pyrimidinedione. Real-time RT-PCR was used to study gene expression. Pyrimidinedione inhibits pneumococcal biofilm growth *in vitro* in a concentration-dependent manner, but it does not inhibit planktonic cell growth. Confocal microscopy analysis revealed the absence of organized biofilms, where cell-clumps were scattered and attached to the bottom of the plate when cells were grown in the presence of pyrimidinedione. Scanning electron microscopy analysis demonstrated the absence of an extracellular polysaccharide matrix in pyrimidinedione-grown biofilms compared to control-biofilms. Pyrimidinedione also significantly inhibited MRSA, MSSA, and *Staphylococcus epidermidis* biofilm growth *in vitro*. Furthermore, pyrimidinedione does not exhibit eukaryotic cell toxicity. In a microarray analysis, 56 genes were significantly up-regulated and 204 genes were significantly down-regulated. Genes involved in galactose metabolism were exclusively up-regulated in pyrimidinedione-grown biofilms. Genes related to DNA replication, cell division and the cell cycle, pathogenesis, phosphate-specific transport, signal transduction, fatty acid biosynthesis, protein folding, homeostasis, competence, and biofilm formation were down regulated in pyrimidinedione-grown biofilms. This study demonstrated that the small molecule Dam inhibitor, pyrimidinedione, inhibits pneumococcal biofilm growth *in vitro* at concentrations that do not inhibit planktonic cell growth and down regulates important metabolic-, virulence-, competence-, and biofilm-related genes. The identification of a small molecule (pyrimidinedione) with *S*. *pneumoniae* biofilm-inhibiting capabilities has potential for the development of new compounds that prevent biofilm formation.

## Introduction

*Streptococcus pneumoniae* (*S*. *pneumoniae*) is an important human pathogen. It causes severe and invasive infections, such as pneumonia, septicemia, otitis media, and meningitis, especially in children, the elderly, and immuno-compromised patients [1,2,3]. *S*. *pneumoniae* initially colonize the nasopharynx and may persist for months without causing illness, forming specialized structures called biofilms [4,5]. Pneumococci from these biofilms can migrate to other sterile anatomical sites, causing severe biofilm-associated infections such as pneumonia and otitis media [6,7,8]. The planktonic bacteria from these biofilm-associated infections can migrate to other sterile sites, such as the blood stream, causing bacteremia, or to the brain, causing meningitis [9,10,11].

A biofilm is defined as a thin layer of bacteria that adhere to each other and to a living tissue or inert surfaces. These bacteria are surrounded by a self-produced polymeric matrix composed of polysaccharides, proteins, and nucleic acids [12]. Bacteria within biofilms possess increased tolerance to antibiotics and are able to resist host defense systems [13,14]. *S*. *pneumoniae* biofilms show increased resistance to common antibiotics, such as penicillin, tetracycline, rifampicin, amoxicillin, erythromycin, clindamycin, levofloxacin, and gentamicin both *in vivo* and *in vitro* [15,16,17]. Bacteria within biofilms exhibit altered physiology, metabolism, and gene expression profiles compared to free-floating planktonic cells [18]. Therefore, existing antimicrobial compounds mainly developed to target planktonic bacteria may not be as effective against biofilms. Moreover, the emergence of antibiotic resistant pneumococcal strains necessitates the identification of alternative drug targets and new antimicrobial compounds that could be effective against pneumococcal biofilms. Effective anti-biofilm strategies could inhibit initial bacterial attachment and colonization, interfere with signaling pathways important for biofilm development, or disrupt the biofilm matrix [19,20,21].

**Fig 1:**
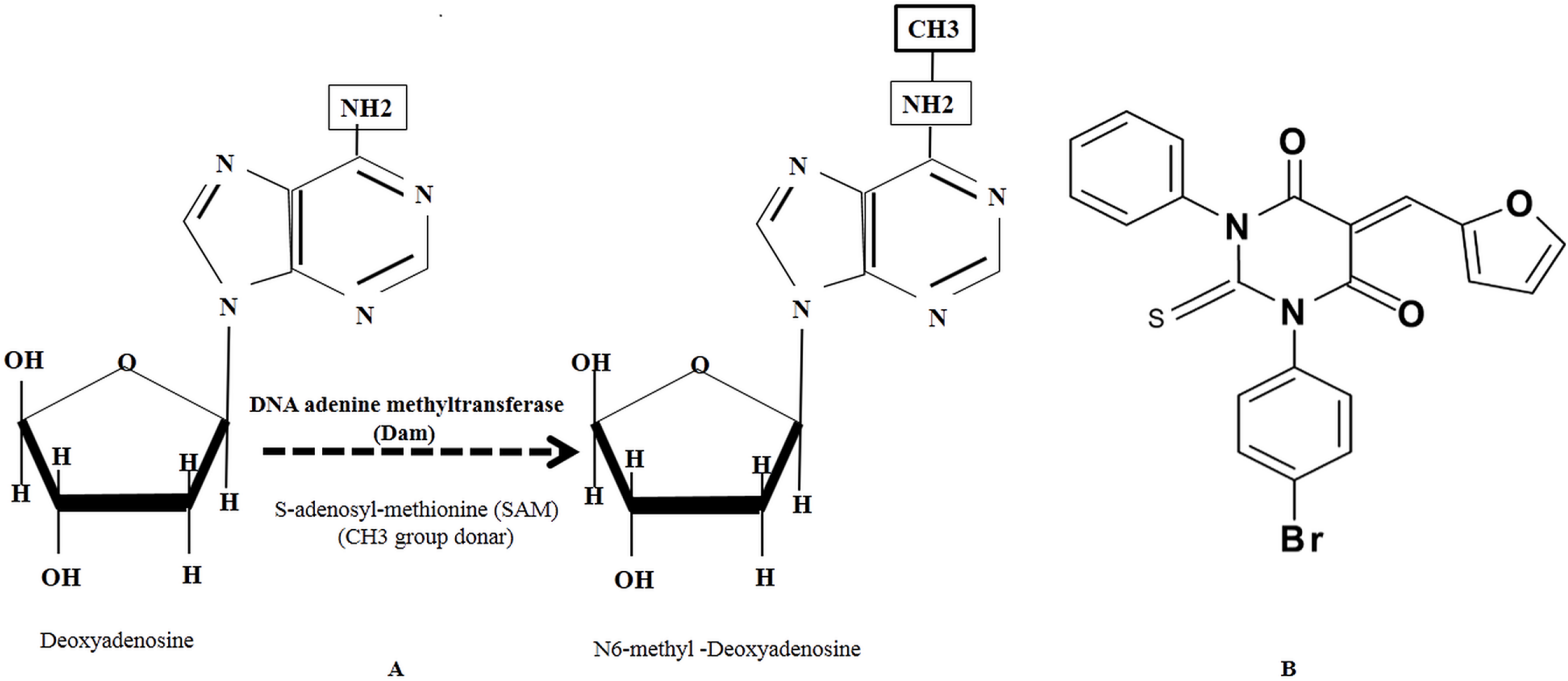
(A) Methyl group transfer from SAM to deoxyadenosine by DNA adenine methyltransferases (Dam). (B) The chemical structure of the small molecule inhibitor, pyrimidinedione.

Bacterial DNA methyltransferases are generally associated with restriction-modification systems, with the exception of DNA adenine methyltransferase (Dam) and cell cycle-regulated methyltransferase (CcrM) [22]. In bacteria, Dam alters the expression of pathogenic genes involved in several cellular activities, including mismatch repair, initiation of chromosomal replication, DNA segregation, and transposition [23,24]. In bacteria the Dam enzyme catalyzes a methyl group transfer from *S*-adenosyl-L-methionine (SAM) to the N^6^ position of adenine in duplex DNA (Fig 1A). This adenine methylation is unique in bacteria. Therefore, these bacterial enzymes represent excellent antimicrobial target candidates. Moreover, SAM-mediated methylation is an important process in pneumococci, leading to the methylation of DNA and macromolecules, as well as the biosynthesis of quorum sensing (QS) molecules and secondary metabolites, such as polyamine, that play roles in biofilm formation [25,26]. Our previous study showed that 5-azacytidine, a hypo-methylating compound, and sinefungin, a SAM analogue, inhibit *S*. *pneumoniae* biofilm growth [27,28]. However, the effect of Dam inhibitor small molecule on pneumococcal biofilm formation has not been studied.

In the present study, we examine the effect of a small molecule Dam inhibitor, pyrimidinedione, on *S*. *pneumoniae* biofilms, evaluating changes in global gene expression via microarray analysis. The small molecule pyrimidinedione,1-(4 bromophenyl)-5-(2-furylmethylene)-3-phenyl-2-thioxodihydro-4, 6 (1H,5H)-pyrimidinedione, was reported to be an effective bacterial Dam and CcrM inhibitor. It binds to the ternary enzyme:DNA:AdoMet complex and prevents Dam activity [29].

Our results demonstrated that pyrimidinedione inhibited pneumococcal biofilm growth *in vitro* at concentrations that did not inhibit planktonic cell growth, and it down-regulated the expression of important metabolic-, virulence-, competence-, and biofilm-related genes. Pyrimidinedione is also effective against MSSA, MRSA, and *Staphylococcus epidermidis* biofilms *in vitro*, and it is not cytotoxic to eukaryotic cells. The identification of a small molecule (pyrimidinedione) with *S*. *pneumoniae* biofilm-inhibiting capabilities has potential for the development of new compounds that prevent biofilm formation.

## Materials and Methods

### Ethics statement

The experimental protocol was approved by the Institutional Review Board of Korea University, Guro Hospital, Seoul, South Korea. The human middle ear epithelium cell (HMEEC) line used in this study was kindly provided by Dr. David J. Lim (House Ear Institute, LA, USA). Pre-made blood agar plates (BAPs) containing 5% v/v sheep blood were purchased from Shin Yang chemicals Co., Ltd. (Seoul, Korea).

### Bacterial strains and culture conditions

*S*. *pneumoniae* serotype 2 (D39 strain; NCTC 7466) was purchased from Health Protection Agency Culture Collections (HPA, Salisbury, UK), serotype 3 (ATCC strain 6303) and serotype 19 (ATCC strain 49619) were purchased from ATCC (Manassas, VA, USA). *S*. *pneumoniae* serotype 11 (strain 7101975) was obtained from the infectious disease department of Korea University Medical Center, Guro Hospital, Seoul). Bacteria were routinely grown in tryptic soy broth (TSB; BD Difco; Detroit, MI, USA) or on BAPs supplemented with 5% v/v sheep blood at 37°C in 5% atmospheric CO_2_. Five methicillin-resistant *Staphylococcus aureus* (MRSA) strains (CCARM 3108, CCARM 3807, CCARM 3912, CCARM 3903, and CCARM 3967) were purchased from Culture Collection of Antimicrobial Resistant Microbes (CCARM; Seoul, Korea). Methicillin-sensitive *Staphylococcus aureus* (MSSA, ATCC 29213) and *Staphylococcus epidermidis* (ATCC 35984) were purchased from ATCC. The small molecule inhibitor (here called pyrimidinedione) 1-(4 bromophenyl)-5-(2-furylmethylene)-3-phenyl-2-thioxodihydro-4, 6 (1H,5H)-pyrimidinedione, was purchased from ChemBridge, USA(catalogue number sc5309471; Fig 1B). A stock solution of pyrimidinedione was prepared in DMSO.

### Growth curve of *S*. *pneumoniae* D39 with pyrimidinedione

Cultures of *S*. *pneumoniae* D39 were grown in the presence of 1 and 10 μM/ml concentrations of pyrimidinedione in a time course experiment. The cell suspensions were incubated at 37°C in 5% CO_2_, and the optical density at 600nm (OD_600_) was measured with a spectrophotometer (SpectraMax plus, Molecular Devices, Sunnyvale, CA, USA) at different time points (1, 2, 3, 4, 5, 6, 7, 8, 9 and 10h). The experiments were performed in replicates of five and were repeated three times to obtain statistical significance.

### Effect of pyrimidinedione on *in vitro* biofilm growth

*S*. *pneumoniae* and Staphylococcus biofilm formation experiments were carried out using a static model in 96-well or 24-well microtiter plates [18,30]. Briefly, bacterial colonies grown overnight on blood agar were scraped and seeded in broth (TSB medium) and further grown until mid-logarithmic phase (1×10^8^ cfu/ml). Bacterial cells were diluted 1:1000 in fresh broth, and 200μl or 1 ml cell suspension was used to inoculate a 96- or 24-well microtiter plate. Plates were then incubated at 37°C in 5% CO_2_. Pyrimidinedione was added to each plate, as indicated. Control samples did not contain any supplement, and DMSO-control samples contained 0.01% DMSO (final concentration). After incubation, the medium and planktonic cells were discarded, and the plates were washed three times with sterile PBS. The plates were air dried for 15 min and stained with crystal violet (CV; 0.1%) for 15 min. The CV stain was removed and the plates were washed again. The stained biofilm was dissolved in 95% ethanol, and the OD_570_ was measured in an automatic spectrophotometer. All experiments were performed with five replicates, and the average was calculated. To quantify the bacteria within biofilms, plates inoculated as described above were washed, and adherent biofilms were dissolved in sterile water via sonication for 10 s. One hundred micro-liter samples were serially diluted, plated on blood agar plates, and incubated at 37°C in 5% CO_2_ for 24 h. After incubation, bacterial colonies were counted and cfu/ml was determined. Planktonic cells (cells in the biofilm supernatant) were counted in a similar manner.

### Effect of pyrimidinedione concentration on biofilm growth

*S*. *pneumoniae* (serotypes 2, 3, 19, and 11) biofilms were grown in different concentrations of pyrimidinedione (0.5–10 μM/ml) for 15 h, as previously described. Biofilm biomass was measured using the CV-microtiter plate method. Cfu counts of bacteria within biofilms (D39 strain) were conducted as described above. The percent decrease in biofilm biomass was calculated by subtracting the biomass of DMSO-control samples. The half-maximal effective concentration (EC_50_) of pyrimidinedione was determined as the concentration corresponding to 50% of the maximum biofilm inhibition with respect to DMSO-control biofilms. At each pyrimidinedione concentration, cells suspended within the biofilm supernatant were collected and analyzed. Bacterial growth was detected by measuring optical density at 600nm (OD_600_), and cfu of planktonic bacteria in the biofilm supernatant of D39 were counted.

To determine whether the biofilm-inhibiting effects of pyrimidinedione extended beyond *S*. *pneumoniae*, pyrimidinedione was tested on other biofilm-forming microbial pathogens, such as MSSA, MRSA, and *S*. *epidermidis*. *Staphylococcus* 24 h biofilm assays were carried out in a similar manner as described above, in TSB medium supplied with 1, 5, and 10 μM/ml pyrimidinedione.

### Effect of pyrimidinedione on biofilms grown at different time points

*In vitro S*. *pneumoniae* biofilm growth varies at different time points. Therefore, we analyzed the effects of pyrimidinedione on *S*. *pneumoniae* D39 biofilms at different time points *in vitro*. *S*. *pneumoniae* biofilm was grown at different concentrations of pyrimidinedione [0 (DMSO control), 0.5, 1, 5, and 10 μM/ml] for 5, 10, 15, and 20 h.

### Effect of pyrimidinedione on established biofilms

To analyze the inhibitory effects of pyrimidinedione on established biofilms, *S*. *pneumoniae* D39 biofilms were grown for 15 h. These established biofilms were then treated with different concentrations (1−400μM/ml) of pyrimidinedione and further incubated at 37°C in 5% CO_2_ for 6 h. The biofilms were washed, and the biofilm biomass was detected by CV-microtiter plate assay.

### Visualization of pneumococcal (D39 strain) biofilm growth by confocal microscopy

*In vitro* biofilm growth, with and without pyrimidinedione, was analyzed by confocal microscopy. Biofilms were grown on microdiscs for 15 h with 7μM/ml pyrimidinedione and stained using LIVE/DEAD Biofilm Viability staining kit (Invitrogen). A control sample was grown with 0.01% DMSO (final concentration). Biofilms were examined with a Nikon A1 confocal microscope (Nikon Instruments Inc., NY, USA) using fluoresce in (green) and Texas red (red) band pass filter sets. The live bacteria with intact cell membranes appear green, and those with damaged membranes appear red.

### Visualization of biofilm morphology by scanning electron microscopy (SEM)

Pneumococcal (D39 strain) biofilms grown with 7μM/ml pyrimidinedione in 24-well tissue culture plates for 15 h were analyzed by SEM. The control sample was grown in 0.01% DMSO. Planktonic cells were removed, and the plates were gently washed twice with sterile PBS (pH 7.4). The samples were pre-fixed for 2 h in a 2% glutaraldehyde and paraformaldehyde solution, followed by 2 h post-fixation in 1% osmic acid. The samples were then treated with a graded series of ethanol (from 60% to 100%), washed three times with t-butyl alcohol, and were then immersed in t-butyl solution at -20°C. The samples were dried in a freeze dryer (ES-2030, Hitachi, Tokyo, Japan) and platinum coated using an IB-5 ion coater (Eiko, Kanagawa, Japan). The samples were visualized using a S-4700 field emission scanning electron microscope (Hitachi).

### Effect of pyrimidinedione on biofilm global gene expression

*In situ* synthesis of oligonucleotide microarrays was used to compare global gene expressions between the *S*. *pneumoniae* D39 strain within biofilms grown in the presence and absence of pyrimidinedione. Biofilms were grown with 7 μM/ml pyrimidinedione, as described above, in 24-well plates and washed with sterile water. The adherent biofilm cells at the bottom and side of plate were scraped, pelleted, and subjected to lysozyme 100 μl (3 mg/ml) treatment for 4 min. Total RNA was isolated using the RNeasy Total RNA Isolation System Kit (Qiagen, Valencia, CA, USA) following the manufacture’s protocol. Contaminated genomic DNA was removed on a column by RNAase-free DNAse (Qiagen) treatment at 20–25°C for 10 min. RNA was quantified by Nano-Drop, and its integrity was checked by capillary electrophoresis using a Bioanalyzer 2100 (Agilent Technologies, Palo Alto, CA, USA).

cRNA probes synthesis and hybridization were performed using a Low Input Quick Amp WT Labeling Kit (Agilent Technologies USA) as per manufacture’s protocol. Briefly, 100ng total RNA was mixed with WT primer mix and incubated for 10 min at 65°C. A cDNA master mix (0.1M DTT, 5X first strand buffer, RNase-Out, 10mM dNTP mix, and MMLV-RT) was prepared separately, and mixed with the RNA with WT primers mix and incubated at 40°C for 2 h and thereafter at 70°C for 10min. Transcription of dsDNA was performed by adding the transcription master mix (NTP mix, 4X transcription buffer, 50% PEG, 0.1 M DTT, RNase-Out, T7-RNA polymerase, inorganic pyrophosphatase, and cyanine 5-CTP) to the dsDNA reaction samples and incubating the samples at 40°C for 2 h. Amplified and labeled cRNA was purified on RNase mini columns (Qiagen) and quantified using a spectrophotometer. The cyanine 5-labeled cRNA mix was fragmented by incubating at 60°C for 30 min with 10X blocking agent and 25X fragmentation buffer. The fragmented cRNA were dissolved in 2X hybridization buffer and pipetted on the assembled *Streptococcus pneumoniae* D39 (MYcroarray.com) 6× 7k Microarray. The hybridization reaction was performed in hybridization oven at 57°C for 17 h and the arrays were washed as per manufacturer’s instructions.

Hybridization images were analyzed with DNA Microarray Scanner (Agilent Technologies) and the data quantification was performed using Agilent Feature Extraction Software version 10.7 (Agilent Technologies). The average fluorescence intensity for each spot was calculated, and the local background was subtracted. All data normalization and the selection of differentially expressed genes were performed using GenoWiz 4.0 (Ocimum Biosolutions, India). Genes were filtered by removing flag-out genes from each experiment. Global normalization was performed. The average normalized signal channel intensities were divided by the average normalized control channel intensities to calculate the average normalization ratio.

Microarray experiments were performed in three biological replicates. Statistical significance was determined by Student’s *t*-test. A *p* value less than 0.05 was considered significant. A 1.4-fold change in each gene, and in each microarray experiment, was considered significant and was included in the final results. The functional annotation (molecular and biological function) and gene ontology of the two sets of differentially expressed genes were determined using the UniPortKB database (http://www.uniport.org/uniport/P0A4M0) and STRING version 9.1. Microarray data were deposited in NCBI's Gene Expression Omnibus (GEO) database (http://www.ncbi.nlm.nih.gov/geo/info/linking.html) and are accessible through GEO Series accession number GSE65339.

### Quantification of gene expression by real-time RT-PCR

Microarray gene expression results were confirmed by real-time RT-PCR. Thirteen differentially expressed genes, along with the control *gyrB* gene, were analyzed by real-time RT-PCR. The primers used are shown in Table 1. cDNA amplification was carried out with real-time PCR in a reaction mixture of 20 μl (total volume). The reaction mixture consisted of 10 μl 2× SYBR Green PCR Master Mix (Roche Applied Science, Indianapolis, IN, USA), 3 pmol of each forward and reverse primers, and 4 μl cDNA. The PCR reaction conditions were as follows: initial denaturation at 95°C for 10 min, followed by 45 cycles of DNA denaturation for 15 s at 95°C, primer annealing for 10 s at 56°C, and extension for 15 s at 72°C; and a final extension step at 72°C for 5 min. To verify that cDNA samples were not contaminated with genomic DNA, a control reaction where no reverse transcriptase was added was included in each RT-PCR experiment. The relative quantification of gene expression was performed using the 2^–ΔΔCT^ method as described elsewhere [31]. Gene expression normalization was performed using a housekeeping gene, *gyrB*, and the standard condition was a biofilm grown without pyrimidinedione.

**Table 1:** List of primers used in this study.

Gene name	Primer sequence	Base-pairs
*purC*	F-GACTGCTTTCAACGGTGTCA	20
	R-ACACCAGCCGCATTTAATTT	20
*capD*	F- AAGCAGGTTTTCTGGGGAAT	20
	R-ACAGGAAGGCCAAACTCGTA	20
*adk*	F- AGGGAACTCAAGCAGCAAAA	20
	R-CAGGAACCAATTCACCCTTG	20
*lacG-2*	F- ACTAGCTGGTTCGGCAGTGT	20
	R-GCTTATCAAGCAGAAGGTGCT	21
*lacT*	F- CAAGCGGAACATCTTTGAGA	20
	R-GATTGCATTCGGAAAAAGGA	20
*galT-1*	F- TGCTCCTAAACATTCCTTTTCC	22
	R- TCCGATGAAAATGACCTGAA	20
*cglD*	F- CTGATGGTGCCTGAATTCCT	20
	R- GAAACCCAAAAACGCAGTGT	20
*nrdG*	F- CAAGAATGGAAAAGCGAGGA	20
	R-AACATCCCTCGCAGTGAAAC	20
*fabD*	F- GGATGGGACGGGATTTCTAT	20
	R- GCGGGTCTGATTGAGTTTGT	20
*dnaK*	F- AAAATCATCGCAAACCCAGA	20
	R- GTGACTGCTTGACGTTTTGC	20
*pstB*	F-TAACCGGATGAACGATTTGG	20
	R-ACCATCCCTACACGCTTACG	20
*phoU*	F-GGGCAACTTGTCCTTGAAAC	20
	R-TTCGATAGCGCTTTGACCTT	20
*acpP*	F-GGACGCAGATTCATTGGACT	20
	R-CGTAAGCAACCAAGTCACCA	20

### Evaluation of pyrimidinedione-mediated eukaryotic cellular cytotoxicity by Cell-Counting Kit (CCK-8)

The cytotoxicity of pyrimidinedione was tested on the human middle ear epithelium cell (HMEEC) line using a CCK-8 kit (Dojindo, MD, USA). HMEECs were kindly provided by Dr. David J. Lim (House Ear Institute, LA, USA) and were maintained in DMEM and BEBM medium (1:1) with required supplements [32,33]. HMEECs (1×10^4^ /well) were seeded in 96-well plates and incubated at 37°C overnight in presence of 5% CO_2_. After overnight culture, HMEECs were treated with pyrimidinedione (1, 5, or 10 μM/ml) for 24 h in triplicate. Positive and negative control wells were supplemented with 0.01% DMSO (final concentration) or 2% Triton X-100, respectively. After incubation, 10 μl CCK8 solution was added to each well, and the cells were incubated for a further 2–3 h. Mitochondrial dehydrogenase within the cells reduced CCK8 solution to a yellow product called formazan. The amount of formazan produced in the reaction sample is positively correlated with cell viability. Absorbance at 450nm was measured using a microplate reader.

### Statistical analysis

Individual experiments were carried out in triplicates or five-replicates, and mean values were calculated. The mean value differences were assessed by Student’s *t*-test, and the statistical significance was set at a *p*-value of less than 0.05.

## Results

### Growth curve of *S*. *pneumoniae* supplemented with pyrimidinedione

**Fig 2:**
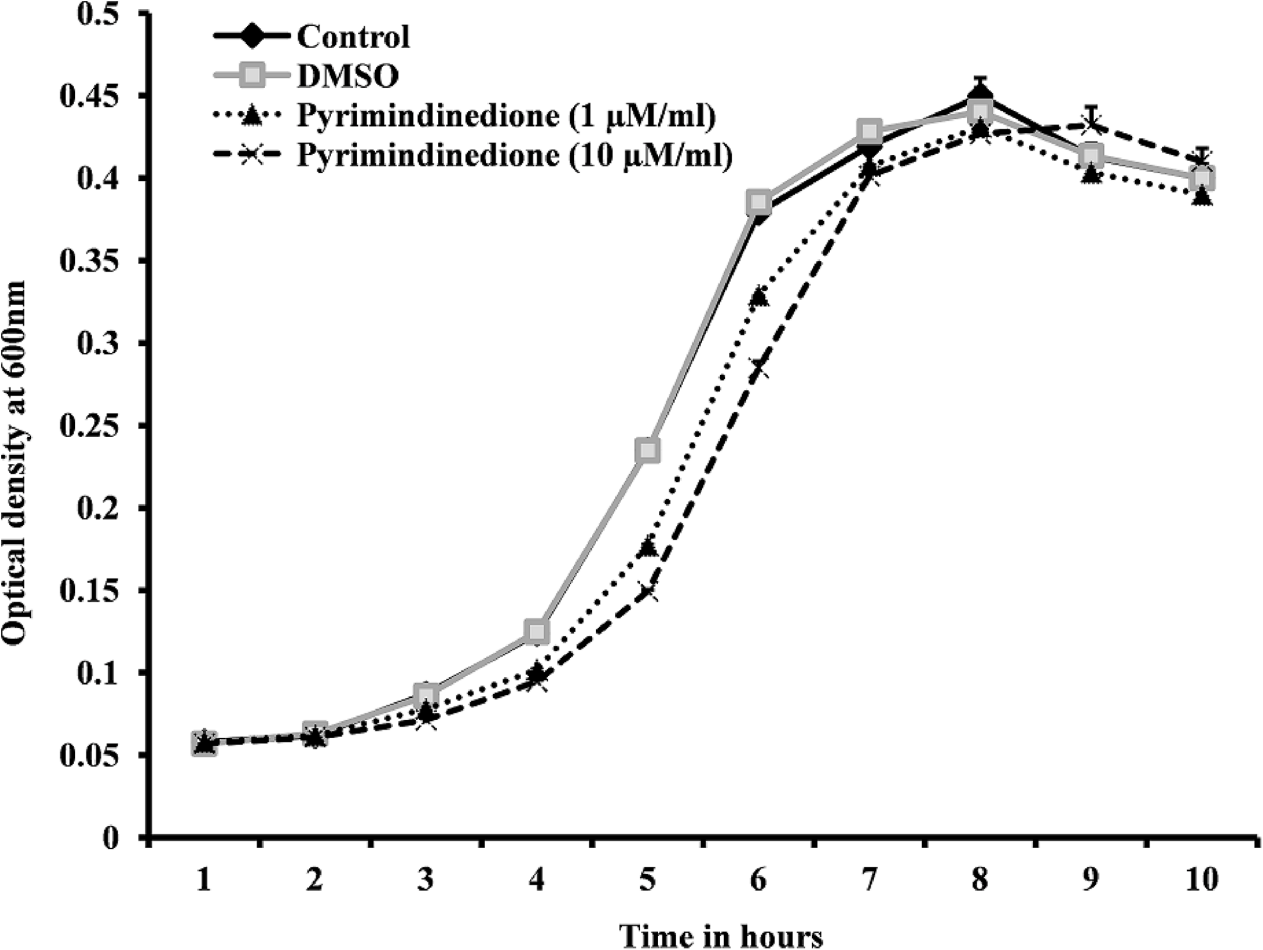
Growth of *Streptococcus pneumoniae* (D39) at different time points and with different concentrations of pyrimidinedione. Pneumococcal cells were incubated at 37°C in 5% CO_2_, and growth was detected by measuring the optical density at 600 nm (OD_600_). Error bars represent the standard deviation of the mean (SD).

The planktonic cell growth of *S*. *pneumoniae* D39 in the presence of pyrimidinedione (1μM/ml or 10 μM/ml) was not significantly inhibited during the time course experiment. At 6 h incubation, 12% of the cells were inhibited, however, at stationary phase (10 h post-inoculation), planktonic cell growth was not inhibited (Fig 2). This result indicated that pyrimidinedione is partially bacteriostatic, and it has no effect on final bacteria planktonic growth.

### Pyrimidinedione inhibits *S*. *pneumoniae* (serotype 2, 3, 19 and 11) biofilm growth *in vitro*

**Fig 3:**
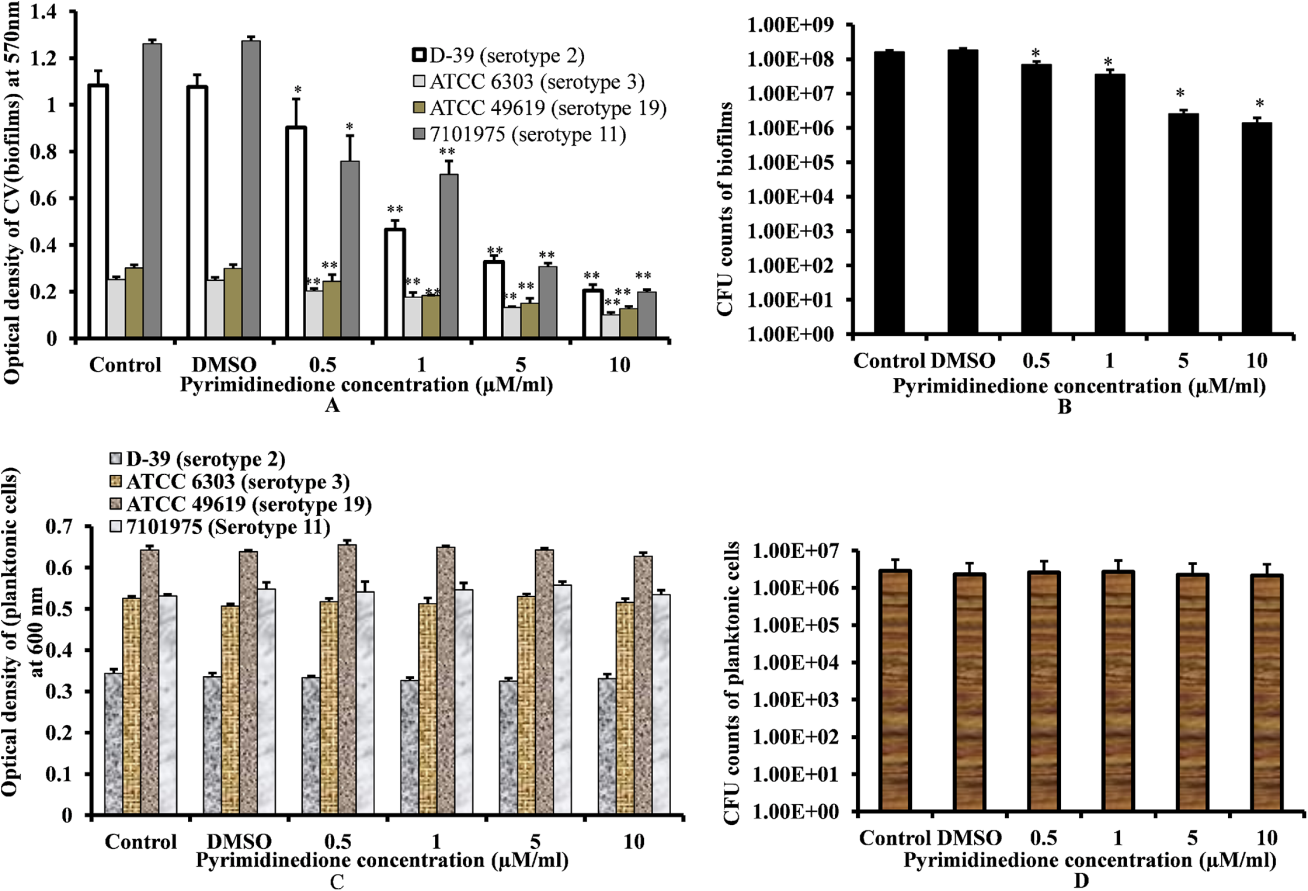
*In vitro S*. *Pneumoniae* biofilm and planktonic cell growth at different pyrimidinedione concentrations at 15 h. (A) Detection of *S*. *pneumoniae* serotypes 2, 3, 19, and 11 biofilm biomasses by CV-microtiter plate assay. (B) Cfu counts of *S*. *pneumoniae* D39 biofilms. (C) Planktonic cell growth detected of *S*. *pneumoniae* (serotype 2, 3, 19 and 11) by measuring optical density at 600 nm. (D) Cfu counts of *S*. *pneumoniae* D39 planktonic cell growth. The results were compared by Student’s *t*-test (*corresponds to *p*< 0.05, **corresponds to *p*< 0.005). The error bars represent the SD.

**Fig 4:**
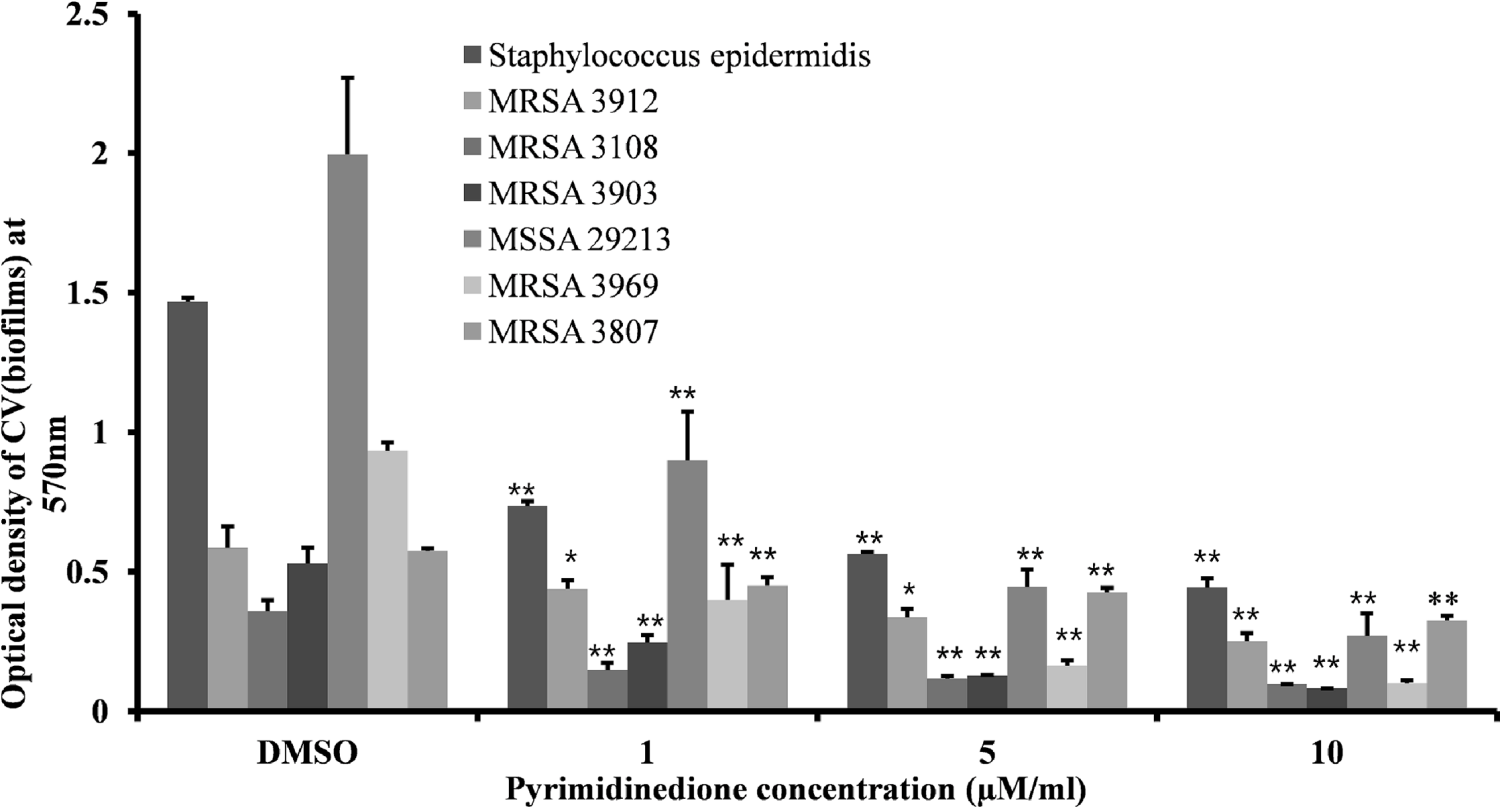
MRSA, MSSA, and *Staphylococcus epidermidis* biofilm growth *in vitro* at different concentrations of pyrimidinedione at 24 h. The biofilm biomass was detected by CV-microtiter plate method. The results were compared by Student’s *t*-test (*corresponds to *p*< 0.05, **corresponds to *p*< 0.005). The error bars represent the SD.

Addition of pyrimidinedione inhibited pneumococcal biofilm growth *in vitro*. A significant decrease (*p*<0.05) in biofilm biomass of *S*. *pneumoniae* serotypes 2, 3, 19, and 11 were detected in biofilms grown in the presence of pyrimidinedione (Fig 3A). The inhibitory effects of pyrimidinedione was concentration dependent, and the calculated EC_50_ (*S*. *pneumoniae* D39) was 1 μM. Addition of 1 μM/ml pyrimidinedione significantly decreased *S*. *pneumoniae* D39 biofilm biomass by 54% (*p*< 0.05, Fig 3A) and cfu counts by 83% (*p*< 0.05, Fig 3B) in comparison to DMSO-control biofilms. However, the planktonic cell growth of *S*. *pneumoniae* serotypes 2, 3, 19, and 11 in the presence of pyrimidinedione was not significantly decreased (Fig 3C). Similarly, no significant decrease in planktonic bacterial cfu counts was detected in the D39 strain (Fig 3D). This result indicated that pyrimidinedione significantly inhibits biofilm growth but has no effect on planktonic growth. The inhibitory effect of pyrimidinedione was significant in all *S*. *pneumoniae* serotypes tested, indicating that the inhibitory effect of pyrimidinedione was independent of serotypes, and the application of this molecule could be extent to other serotypes biofilms as well. Pyrimidinedione significantly inhibited *in vitro* MSSA, MRSA, and *S*. *epidermidis* biofilm growth in a dose-dependent manner, similar to that observed in pneumococci (Fig 4). *S*. *epidermidis*, MSSA 29213 and MRSA CCARM 3903 are strong biofilm producing strains. 1 μM/ml concentration of pyrimidinedione significantly (*p*< 0.05) decreased biofilm biomass of *S*. *epidermidis*, MSSA 29213 and MRSA 3903 by 50, 55 and 53% respectively. This indicates that pyrimidinedione is equally effective against both antibiotic resistance and sensitive strong biofilm producing *staphylococcus*.

### Pyrimidinedione inhibits *S*. *pneumoniae* D39 biofilm growth at both early and late stages

**Fig 5:**
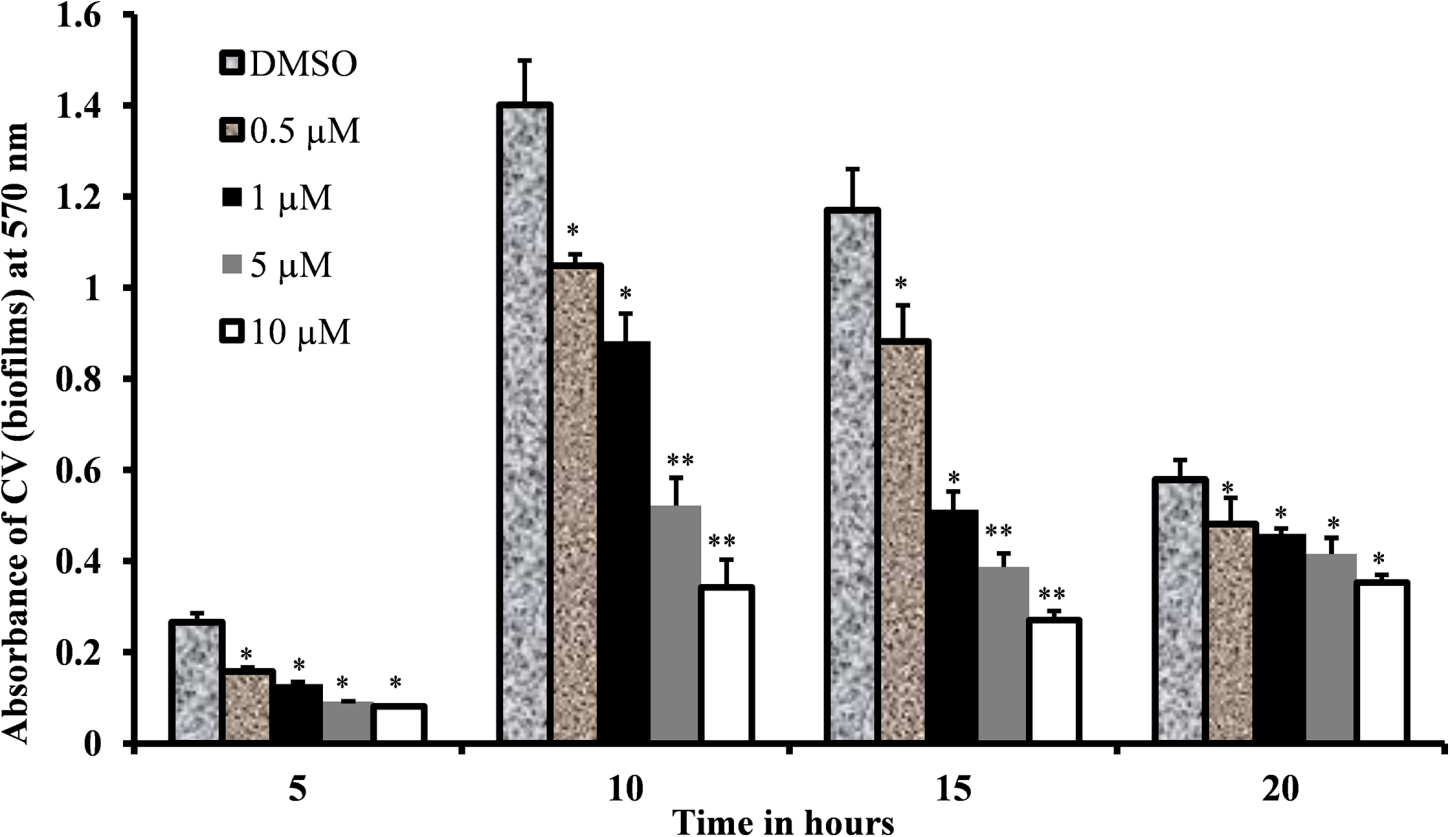
*S*. *pneumoniae* D39 biofilms grown at different concentrations of pyrimidinedione over time. Biofilm biomass was detected by CV-microtiter plate assay. The results were compared by Student’s *t*-test (* corresponds to *p*< 0.05, ** corresponds to *p*< 0.01). The error bars represent the SD.

The growth of biofilms under different pyrimidinedione conditions was inhibited at each time point analyzed. A dose-dependent decrease in biofilm biomass was detected in biofilms grown with pyrimidinedione compared to DMSO-control samples (Fig 5). In the DMSO control, there was an increase in biofilm growth at 5 h of incubation, maximal growth occurred at 10 h of incubation, and then biofilm growth declined. A 70% decrease in biofilm growth was observed with the addition of 5 μM pyrimidinedione at 10 h of incubation. These results indicated that pyrimidinedione is an effective inhibitor of *S*. *pneumoniae* biofilm growth *in vitro* at both early (5 h) and late (20 h) stages.

### Pyrimidinedione has no effect on established biofilm biomass

**Fig 6:**
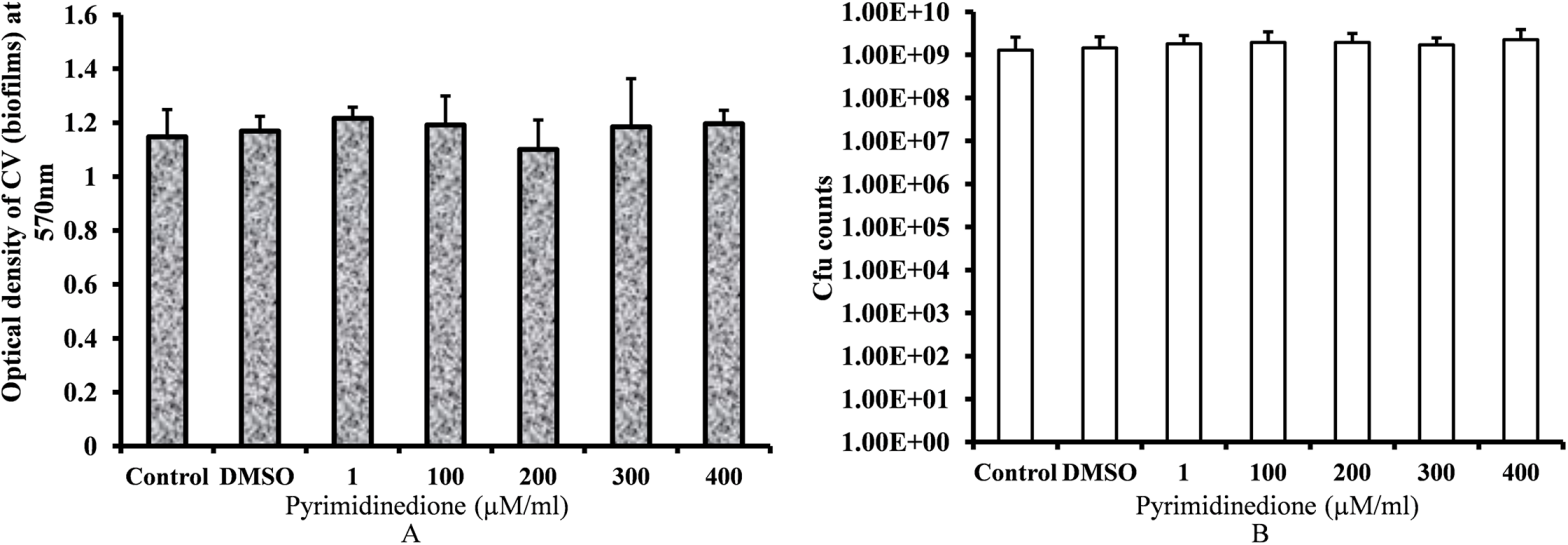
Effects of pyrimidinedione on established biofilms. Established *S*. *pneumoniae* D39 biofilms were treated with different concentrations of pyrimidinedione. (A) Biofilm biomasses were measured by CV-microtiter plate assay. (B) Cfu counts of biofilms. The error bars represent the SD.

Established *S*. *pneumoniae* biofilms were treated with different concentrations of pyrimidinedione. Biofilm quantification by CV-microtiter plate assay demonstrated no significant decrease in biofilm biomass following a 6-h treatment with pyrimidinedione (Fig 6A). There was also no difference observed in cfu counts between control and pyrimidinedione-treated biofilms (Fig 6B). This indicates that pyrimidinedione is unable to dismantle biofilms or kill bacteria within biofilms. Hence, this small molecule cannot eradicate an established biofilm.

### Visualization of biofilm growth by confocal microscopy

**Fig 7:**
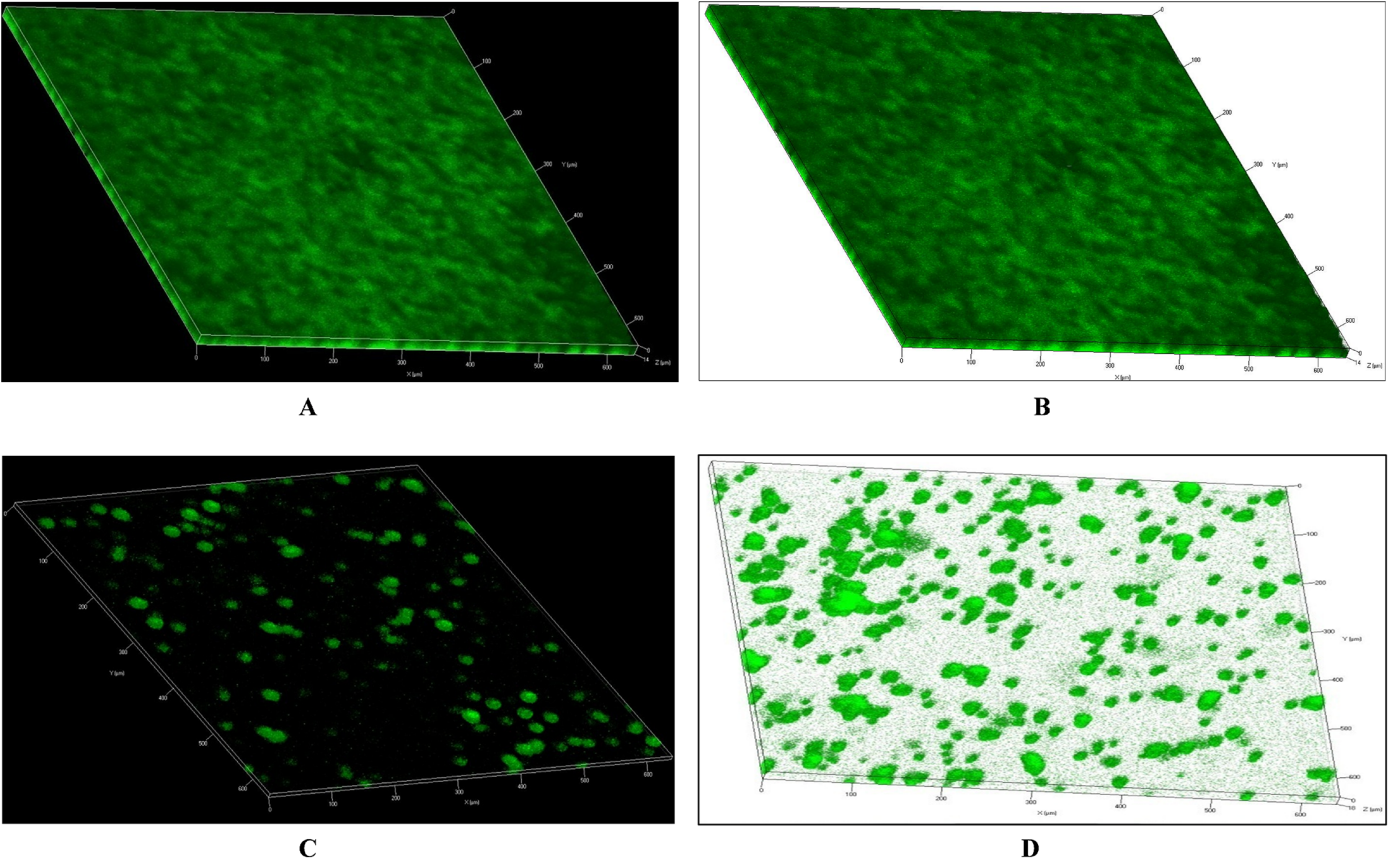
Confocal microscopic images of *Streptococcus pneumoniae* D39 biofilms grown with and without pyrimidinedione. **(**A & B) Representative confocal images of a control sample. The biofilms in the control sample were thick with an organized 3-dimesional structure. **(**C & D) Representative confocal images of biofilms grown with 7 μM/ml pyrimidinedione. The pyrimidinedione-grown biofilms were thin and disorganized, with clumps of cells attached to the bottom of plate.

Confocal microscopy analysis revealed a significant difference in the morphology of biofilms grown in the presence of pyrimidinedione compared to control biofilms (DMSO alone). Control biofilms were compact, thick, and had a well-organized three-dimensional structure (Fig 7A & 7B). In contrast, biofilms grown with pyrimidinedione were thin, the cells were scattered and attached to the bottom of disc, and their three-dimensional structure was not well organized (Fig 7B & 7D). This strongly suggested that pyrimidinedione inhibits biofilm growth and the formation of organized structures.

### Visualization of biofilm morphology by scanning electron microscopy

**Fig 8:**
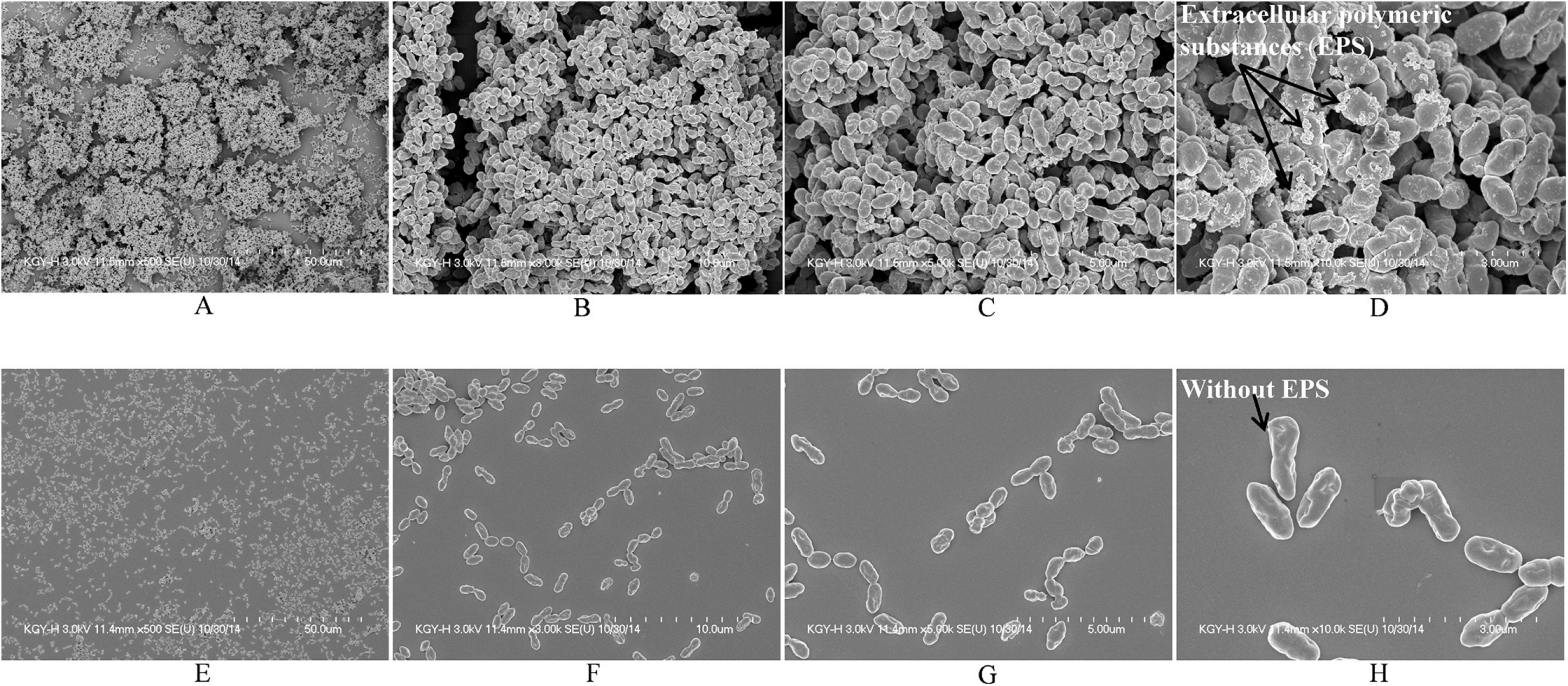
SEM images of *Streptococcus pneumoniae* D39 biofilms grown with and without pyrimidinedione. (A, B, C & D) Representative SEM images of control biofilms. (E, F, G & H) Representative SEM images of biofilms grown with 7 μM/ml pyrimidinedione. The SEM image scale bar corresponds to 50, 10, 5, and 3 μm (from left to right.)

SEM analysis revealed that control biofilms (DMSO-control) were thick, organized, and heterogeneous with micro-colonies. The cells were surrounded by extracellular matrix and were connected to the bottom of the plate and to each other, forming a three-dimensional organized biofilm structure of significant depth (Fig 8A, 8B, 8C & 8D). The extracellular polysaccharide matrix (EPS) was clearly visible on the cell surface (Fig 8D, arrow). In contrast, pyrimidinedione-exposed biofilms were thin, disorganized, and devoid of micro-colonies. Cells were attached only to the base of the plate, while cell-cell adherence was absent (Fig 8E, 8F, 8G & 8H). The cell surfaces were smooth and devoid of matrix and EPS. These results indicated that in presence of pyrimidinedione, pneumococci were unable to form an organized biofilm.

### Effect of pyrimidinedione on biofilm global gene expression

**Fig 9:**
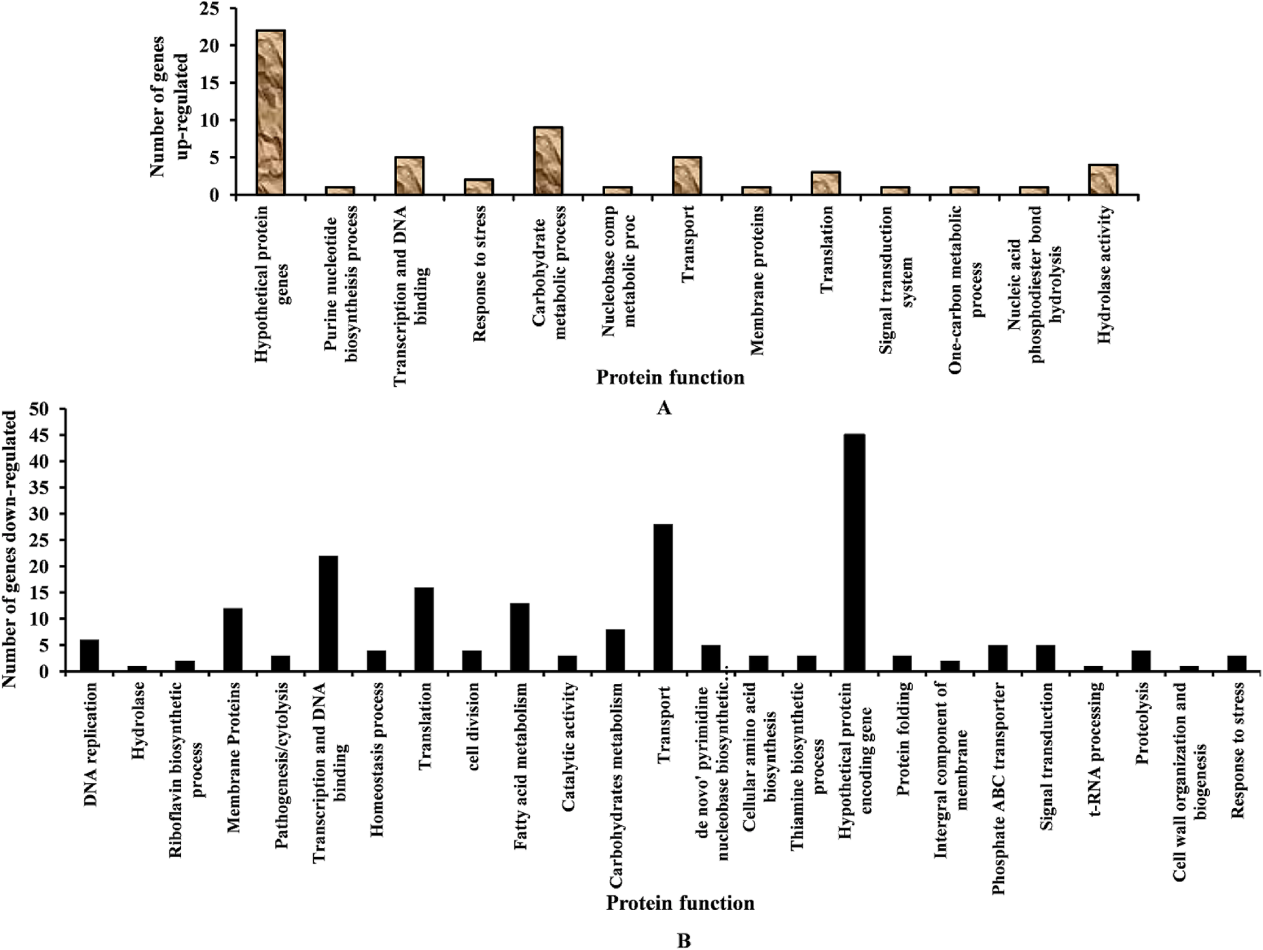
Differential gene expression detected by microarray in pyrimidinedione-grown biofilms with respect to control biofilms. (A) Number of genes significantly (*p*< 0.05) up-regulated. (B) Number of genes significantly (*p*< 0.05) down-regulated in pyrimidinedione-grown biofilms.

Upon microarray analysis, it was determined that the expression of 56 *S*. *pneumoniae* genes was significantly (*p*< 0.05) up-regulated, while expression of 204 genes was significantly (*p*< 0.05) down-regulated in pyrimidinedione-grown biofilms, as compared to controls. Among the 56 up-regulated genes, 22 encoded uncharacterized and hypothetical proteins, and 34 encoded functional proteins (Table 2). Of the 204 down-regulated genes, 45 encoded hypothetical proteins and 159 encoded functional proteins (Table 3). Genes involved in some functional protein categories were exclusively down-regulated or up-regulated in pyrimidinedione-grown biofilms (Fig 9).

**Table 2:** Genes up-regulated in pneumococcal biofilms grown with pyrimidinedione.

Gene locus/name	Protein	Molecule function	Biological process	Mean fold change in expression (p-value)
**Purine nucleotide biosynthetic process**				
SPD_0051 (purC)	phosphoribosylaminoimidazole-succinocarboxamide	ATP binding/phosphoribosylaminoimidazolesuccinocarboxamide synthase activity	'de novo' IMP biosynthetic process	2.3 0.03)
**Carbohydrate metabolism**				
SPD_0940	UDP-N-acetyl-D-mannosaminuronic acid	NAD bindingSource: InterPro/ 3. oxidoreductase activity, acting on the CH-OH group of donors, NAD or NADP as acceptor	polysaccharide biosynthetic process	4.8 (0.01)
SPD_0236 (talc)	transaldolase, putative	sedoheptulose-7-phosphate:D-glyceraldehyde-3-phosphate glyceronetransferase activity	pentose-phosphate shunt	1.4 (0.01)
SPD_1046 (lacG-2)	6-phospho-beta-galactosidase	6-phospho-beta-galactosidase activity	lactose catabolic process via tagatose-6-phosphate	5.3 (0.01)
SPD_1053 (lacA)	galactose-6-phosphate isomerase, LacA subunit	galactose-6-phosphate isomerase activity	galactose catabolic process	1.4 (0.04)
SPD_1050 (lacD)	tagatose 1,6-diphosphate aldolase	tagatose-bisphosphate aldolase activity	lactose catabolic process via tagatose-6-phosphate	1.4 (0.05)
SPD_1051 (lacC)	tagatose-6-phosphate kinase	tagatose-6-phosphate kinase activity	lactose catabolic process via tagatose-6-phosphate	1.4 (0.05)
SPD_1613 (galT-1)	galactose-1-phosphate uridylyltransferase	UDP-glucose:hexose-1-phosphate uridylyltransferase activity	galactose metabolic process	3.5 (0.03)
SPD_1612 (galE-2)	UDP-glucose 4-epimerase	coenzyme binding	galactose metabolic process	1.4 (0.01)
SPD_1052 (lacB)	galactose-6-phosphate isomerase, LacB subunit	galactose-6-phosphate isomerase activity	galactose catabolic process	1.4 (0.01)
**Transport**				
SPD_0234	PTS system, IIC component	protein-N(PI)-phosphohistidine-sugar phosphotransferase activity	phosphoenolpyruvate-dependent sugar phosphotransferase system	1.4 (0.04)
SPD_1048 (lacF-2)	PTS system, lactose-specific IIA component	transferase activity	phosphoenolpyruvate-dependent sugar phosphotransferase system	2.6 (0.01)
SPD_1047 (lacE-2)	PTS system, lactose-specific IIBC components	protein-N(PI)-phosphohistidine-lactose phosphotransferase system transporter activity	phosphoenolpyruvate-dependent sugar phosphotransferase system	3.9 (0.03)
clgD (SPD_1860)	competence protein CglD	Competence-related DNA transformation transporter (DNA-T) core components	Competence-related DNA transformation transporter (DNA-T) core components	1.7 (0.03)
potD (SPD_1218)	spermidine/putrescine ABC transporter,	polyamine binding	polyamine transport	1.4 (0.04)
**Response stress**				
SPD_0286	glutathione peroxidase	glutathione peroxidase activity	response to oxidative stress	1.3 (0.04)
SPD_1287 (trxB)	thioredoxin-disulfide reductase	flavin adenine dinucleotide binding	removal of superoxide radicals	1.5 (0.05)
**Transcription and DNA binding**				
SPD_0280	transcriptional regulator, putative	protein-N(PI)-phosphohistidine-sugar phosphotransferase activity	regulation of transcription, DNA-templated	1.4 (0.05)
SPD_1798	DNA-binding response regulator	DNA binding/sequence-specific DNA binding transcription factor activity	transcription, DNA-templated/phosphorelay signal transduction system	1.5 (0.03)
SPD_1947	transcriptional regulator, putative	sequence-specific DNA binding		3.5 (0.05)
SPD_0352	DNA-binding response regulator	sequence-specific DNA binding transcription factor activity	transcription, DNA-templated	1.4 (0.05)
SPD_1049 (lacT)	transcription antiterminator LacT	RNA binding	regulation of transcription, DNA-templated	3.4 (0.01)
**Nucleic acid phosphodiester bond hydrolysis**				
SPD_0662	endonuclease/exonuclease/phosphatase family	endonuclease activity	Not available	1.5 (0.01)
Translation				
SPD_0494 (valS)	valyl-tRNA synthetase	aminoacyl-tRNA editing activity	valyl-tRNA aminoacylation	1.7 (0.05)
SPD_0757 (rpsA)	ribosomal protein S1	RNA binding	translation	1.4 (0.06)
SPD_0777 (thiI)	thiamine biosynthesis/tRNA modification protein	tRNA adenylyltransferase activity	thiamine biosynthetic process	1.4 (0.02)
**Hydrolase activity**				
SPD_1061	serine/threonine protein phosphatase	hydrolase activity		1.4 (0.04)
SPD_1105 (Rnc)	ribonuclease III	ribonuclease III activity/rRNA binding/Endonuclease, Hydrolase, Nuclease	mRNA processing, rRNA processing, tRNA processing	1.4 (0.01)
SPD_0266	Cof family protein	hydrolase activity	Not available	1.4 (0.02)
SPD_1180	CAAX amino terminal protease family protein	peptidase activity		3.5 (0.04)
**One carbon metabolic process**				
SPD_1087 (Fhs)	formate—tetrahydrofolate ligase	formate-tetrahydrofolate ligase activity	folic acid-containing compound biosynthetic process	1.5 (0.03)
**Membrane protein**				
SPD_1213	membrane protein, putative			1.7 (0.05)
**Signal transduction system**				
SPD_1799	sensor histidine kinase, putative	phosphorelay sensor kinase activity		1.4 (0.04)
**Nucleobase-containing compound metabolic process**				
SPD_0214 (Adk)	adenylate kinase	adenylate kinase activity	AMP salvage	2.7 (0.05)
**Hypothetical proteins**				
SPD_1945	membrane protein, putative			3.0 (0.02)
SPD_0056	vanZ protein, putative			2.9 (0.04)
SPD_0935	Tn5252, Orf 9 protein			3.0 (0.05)
SPD_0023	conserved hypothetical protein			2.2 (0.05)
SPD_0094	conserved hypothetical protein			1.4 (0.02)
SPD_0668	conserved hypothetical protein			2.2 (0.05)
SPD_0796	conserved hypothetical protein			1.5 (0.03)
SPD_0829	conserved hypothetical protein			4.0 (0.05)
SPD_0831	conserved domain protein			7.3 (0.03)
SPD_0923	conserved hypothetical protein			5.2 (0.04)
SPD_0924	conserved hypothetical protein			3.5 (0.05)
SPD_1045	hypothetical protein			6.1 (0.02)
SPD_1848	conserved hypothetical protein			1.4 (0.05)
SPD_1943	conserved hypothetical protein			2.5 (0.04)
SPD_1319	conserved hypothetical protein			1.6 (0.01)
SPD_1261	conserved hypothetical protein			4.3 (0.006)
SPD_1378	conserved hypothetical protein			2.2 (0.001)
SPD_1281	conserved hypothetical protein			1.5 (0.02)
SPD_1417	conserved hypothetical protein			1.8 (0.03)
SPD_1746	conserved hypothetical protein			2.3 (0.05)
SPD_1946	conserved hypothetical protein			3.1 (0.03)
SPD_0981	adenylate cyclase, putative			2.3 (0.02)

**Table 3:** Gene down-regulated in pneumococcal biofilms grown with pyrimidinedione.

Gene locus/name	Protein	Molecular function	Biological function	Mean fold gene expression (p-value)
**DNA replication**				
SPD_0002 (dnaN)	DNA polymerase III, beta subunit	3'-5' exonuclease activity/DNA-directed DNA polymerase activity	DNA replication	-1.5 (0.009)
SPD_0760 (dnaX)	DNA polymerase III, gamma and tau subunits	ATP binding/DNA-directed DNA polymerase activity	DNA replication	-2.6 (0.01)
SPD_2054 (recF)	recF protein	ATP binding	DNA replication/Repair	-1.4 (0.002)
**Integral component of membrane**				
SPD_0040	membrane protein, putative	integral component of membrane		-1.5 (0.004)
SPD_0523 (vex3)	ABC transporter, transmembrane protein Vexp3		Integral component of membrane	-1.5 (0.003)
**Transcription and DNA binding**				
SPD_0064	transcriptional regulator, GntR family protein	DNA binding /sequence-specific DNA binding transcription factor activity	transcription, DNA-templated	-1.5 (0.01)
SPD_0379	transcriptional regulator, MarR family protein	DNA binding	Transcription	-2.0 (0.03)
SPD_0447	transcriptional regulator, MerR family protein	DNA binding	regulation of transcription, DNA-templated	-1.6 (0.03)
SPD_0458 (hrcA)	heat-inducible transcription repressor HrcA	DNA binding	transcription, DNA-templated	-1.4 (0.04)
SPD_0479 (nusA)	transcription termination factor NusA	RNA binding/sequence-specific DNA binding transcription factor activity	Regulation of DNA-templated transcription, termination	-1.6 (0.05)
SPD_1134 (pyrR)	pyrimidine operon regulatory protein/uracil	RNA binding/uracil phosphoribosyltransferase activity	DNA-templated transcription, termination	-1.4 (0.001)
SPD_1523	transcriptional regulator, NrdR family protein	DNA binding	negative regulation of transcription, DNA-templated	-1.5 (0.02)
SPD_1547	DNA-directed RNA polymerase omega chain,	DNA-directed RNA polymerase activity	transcription, DNA-templated	-1.6 (0.01)
SPD_0081	DNA-binding response regulator	DNA binding	transcription, DNA-templated	-1.5 (0.007)
SPD_1758 (rpoC)	DNA-directed RNA polymerase, beta' subunit	DNA-directed RNA polymerase activity	transcription, DNA-templated	-1.5 (0.01)
SPD_1797 (ccpA)	catabolite control protein A	sequence-specific DNA binding transcription factor activity	transcription, DNA-templated	-1.4 (0.01)
SPD_1819 (nusG)	transcription termination/antitermination factor	DNA-templated transcription, elongation		-1.7 (0.04)
SPD_1818 (comX2)	transcriptional regulator ComX2	sequence-specific DNA binding transcription factor activity	DNA-templated transcription, initiation	-1.4 (0.002)
SPD_0467 (blpS)	BlpS protein	DNA binding		-1.5 (0.04)
SPD_1594	transcriptional regulator	sequence-specific DNA binding	DNA binding	-1.9 (0.001)
SPD_1236 (spx)	regulatory protein Spx			-1.4 (0.05)
SPD_0691	transcriptional regulator, PadR family protein			-1.8 (0.004)
SPD_0908	Sua5/YciO/YrdC/YwlC family protein	double-stranded RNA binding		-1.6 (0.01)
SPD_1014	IS630-Spn1, transposase Orf1	DNA binding		-1.6 (0.01)
SPD_1594	transcriptional regulator	sequence-specific DNA binding	DNA binding	-1.9 (0.001)
SPD_0716	IS630-Spn1, transposase Orf1	DNA binding		-1.6 (0.05)
SPD_1708	IS1167, transposase	DNA binding	transposition, DNA-mediated	-1.4 (0.01)
SPD_1521 (dnaI)	primosomal protein DnaI	ATP binding		-1.4 (0.03)
SPD_0315 (cps2A)	integral membrane regulatory protein Cps2A		DNA replication	-2.0 (0.03)
**Transport**				
SPD_0069	PTS system, IIA component		phosphoenolpyruvate-dependent sugar phosphotransferase system	-2.6 (0.01)
SPD_0076	potassium uptake protein, Trk family protein	cation transmembrane transporter activity		-1.5 (0.01)
SPD_0224	iron(III) ABC transporter, permease protein		transport	-1.6 (0.01)
SPD_0424	PTS system, cellobiose-specific IIC component	protein-N(PI)-phosphohistidine-sugar phosphotransferase activity	phosphoenolpyruvate-dependent sugar phosphotransferase system	-1.5 (0.03)
SPD_1141 (uraA)	uracil-xanthine permease	transporter activity	transmembrane transport	-1.4 (0.05)
SPD_1425	transporter, major facilitator family protein	transporter activity	transmembrane transport	-4.2 (0.03)
SPD_1170	oligopeptide ABC	transporter activity		-2.0 (0.04)
SPD_0887	amino acid permease family protein	amino acid transmembrane transporter activity		-1.4 (0.05)
SPD_1425	transporter, major facilitator family protein	transporter activity	transmembrane transport	-4.2 (0.03)
SPD_1409	sugar ABC transporter, ATP-binding protein	hydrolase activity, acting on acid anhydrides, catalyzing transmembrane movement of substances		-1.4 (0.02)
SPD_1820 (secE)	preprotein translocase, SecE subunit	P-P-bond-hydrolysis-driven protein transmembrane transporter activity	protein secretion	-1.7 (0.006)
SPD_1847	PTS system, membrane component, putative		phosphoenolpyruvate-dependent sugar phosphotransferase system	-1.6 (0.02)
SPD_1832	PTS system, IIB component	protein-N(PI)-phosphohistidine-sugar phosphotransferase activity	phosphoenolpyruvate-dependent sugar phosphotransferase system	-1.4 (0.05)
SPD_1831	PTS system, IIC component	protein-N(PI)-phosphohistidine-sugar phosphotransferase activity	phosphoenolpyruvate-dependent sugar phosphotransferase system	-1.7 (0.04)
SPD_1833	PTS system, IIA component	transferase activity	phosphoenolpyruvate-dependent sugar phosphotransferase system	-1.6 (0.01)
SPD_1934 (malX)	maltose/maltodextrin ABC transporter,	maltose transmembrane transporter activity		-1.4 (0.01)
SPD_2026	ABC transporter, permease protein	transport		-1.6 (0.01)
SPD_0400	Glycosyl transferase family protein 8, putative	transferase activity, transferring glycosyl groups		-1.5 (0.05)
SPD_1677 (rafE)	sugar ABC transporter, sugar-binding protein	transporter activity		-1.9 (0.05)
SPD_1755	ABC transporter, ATP-binding protein	ATPase activity		-1.9 (0.02)
SPD_1738 (dinF)	MATE efflux family protein DinF	drug transmembrane transporter activity		-2.2 (0.05)
SPD_1528	ABC transporter, ATP-binding protein	ATPase activity		-1.7 (0.002)
SPD_1438	cadmium resistance transporter, putative			-1.4 (0.01)
SPD_1431	glycosyl transferase, group 2 family protein	transferase activity, transferring glycosyl groups		-1.4 (0.04)
SPD_1383	cation-transporting ATPase, E1-E2 family			-1.5 (0.03)
SPD_1176	ABC transporter, ATP-binding protein	ATPase activity/ATP binding		-1.9 (0.02)
SPD_0960 (cpoA)	glycosyl transferase CpoA	transferase activity, transferring glycosyl groups	biosynthetic process	-2.0 (0.01)
SPD_0189	acetyltransferase, GNAT family protein	N-acetyltransferase activity		-2.3 (0.03)
**Phosphate ABC transporter**				
SPD_1912 (pstA)	phosphate ABC transporter, permease protein	inorganic phosphate transmembrane transporter activity	phosphate ion transmembrane transport	-1.7 (0.01)
SPD_1910 (pstS)	phosphate ABC transporter, phosphate-binding	ABC transporters	Signal transduction	-1.8 (0.05)
SPD_1913 (pstB)	phosphate ABC transporter, ATP-binding protein	phosphate ion transmembrane-transporting ATPase activity		-1.9 (0.008)
SPD_1914 (phoU)	phosphate transport system regulatory protein	phosphate ion transport		-2.2 (0.01)
SPD_1911 (pstC)	phosphate ABC transporter, permease protein	inorganic phosphate transmembrane transporter activity	phosphate ion transport	-1.6 (0.01)
**Signal transduction**				
SPD_0082	sensor histidine kinase	ATP binding/phosphorelay sensor kinase activity		-1.4 (0.001)
SPD_0701 (ciaR)	DNA-binding response regulator CiaR			-1.4 (0.002)
SPD_0702 (ciaH)	sensor histidine kinase CiaH	ATP binding/phosphorelay sensor kinase activity		-1.5 (0.003)
SPD_2065 (comC1)	competence-stimulating peptide type 1	Two-component system		-1.5 (0.005)
SPD_1040 (ptsH)	phosphocarrier protein HPr	protein serine/threonine kinase activity	phosphoenolpyruvate-dependent sugar phosphotransferase system	-1.6 (0.005)
**Membrane protein**				
SPD_0080	cell wall surface anchor family protein		Cell wall component	-1.7 (0.005)
SPD_0162	membrane protein, putative			-2.5 (0.05)
SPD_0282	membrane protein, putative			-1.8 (0.05)
SPD_1237	membrane protein, putative			-4.6 (0.009)
SPD_1265	membrane protein, putative			-1.5 (0.008)
SPD_1422	membrane protein, putative	Membrane protein		-1.5 (0.006)
SPD_1426	membrane protein, putative			-1.9 (0.04)
SPD_1175	membrane protein, putative			-1.4 (0.02)
SPD_1717	membrane protein, putative			-2.6 (0.01)
SPD_1589	lipoprotein, putative			-2.3 (0.01)
SPD_1527	membrane protein, putative			-2.1 (0.04)
SPD_1965 (pcpA)	choline binding protein PcpA			-1.7 (0.003)
**t-RNA processing**				
SPD_0129 (gidA)	tRNA uridine 5-carboxymethylaminomethyl	flavin adenine dinucleotide binding	tRNA wobble uridine modification	-2.1 (0.05)
**Proteolysis**				
SPD_0258 (pepS)	aminopeptidase PepS	aminopeptidase activity		-1.8 (0.01)
SPD_0308 (clpL)	ATP-dependent Clp protease, ATP-binding subunit	ATP binding/peptidase activity		-1.5 (0.006)
SPD_0558 (prtA)	cell wall-associated serine protease PrtA	serine-type endopeptidase activity		-1.9 (0.04)
SPD_0577 (zmpB)	zinc metalloprotease ZmpB	metalloendopeptidase activity/zinc ion binding		-1.4 (0.03)
**Riboflavin biosynthetic process**				
SPD_0167 (ribB)	3,4-dihydroxy-2-butanone 4-phosphate	3,4-dihydroxy-2-butanone-4-phosphate synthase activity/GTP binding	riboflavin biosynthetic process	-2.2 (0.007)
SPD_0168 (ribE)	riboflavin synthase, alpha subunit	oxidoreductase activity/riboflavin synthase activity	riboflavin biosynthetic process	-1.7 (0.006)
**Translation**				
SPD_0192 (rpsJ)	ribosomal protein S10	structural constituent of ribosome/tRNA binding	translation	-1.5 (0.01)
SPD_0194 (rplD)	ribosomal protein L4	rRNA binding	translation	-1.4 (0.003)
SPD_0197 (rpsS)	ribosomal protein S19	rRNA binding/structural constituent of ribosome	translation	-1.4 (0.01)
SPD_0198 (rplV)	ribosomal protein L22	rRNA binding/structural constituent of ribosome	translation	-1.7 (0.02)
SPD_0199 (rpsC)	ribosomal protein S3	rRNA binding	translation	-1.4 (0.006)
SPD_0201 (rpmC)	ribosomal protein L29	structural constituent of ribosome	translation	-1.5 (0.01)
SPD_0202 (rpsQ)	ribosomal protein S17	rRNA binding/structural constituent of ribosome	translation	-1.9 (0.04)
SPD_0204 (rplX)	ribosomal protein L24	rRNA binding/structural constituent of ribosome	translation	-1.7 (0.03)
SPD_0083 (rpsD)	ribosomal protein S4	rRNA binding/structural constituent of ribosome	translation	-1.4 (0.005)
SPD_0835 (frr)	ribosome recycling factor		translational termination	-1.7 (0.03)
SPD_1148 (rplS)	ribosomal protein L19	structural constituent of ribosome	translation	-1.6 (0.002)
SPD_0906 (prfA)	peptide chain release factor 1	translation release factor activity, codon specific		-2.9 (0.03)
SPD_1245 (rpsU)	ribosomal protein S21			-1.6 (0.05)
SPD_1370 (rpsF)	ribosomal protein S6			-1.4 (0.03)
SPD_0481	ribosomal protein L7A family protein	Ribonucleoprotein, Ribosomal protein		-1.7 (0.01)
SPD_2033 (yfiA)	ribosomal subunit interface protein			-2.2 (0.006)
**Fatty acid biosynthetic process**				
SPD_0380 (fabH)	3-oxoacyl-(acyl-carrier-protein) synthase III	3-oxoacyl-[acyl-carrier-protein] synthase activity/beta-ketoacyl-acyl-carrier-protein synthase III activitY	fatty acid biosynthetic process	-1.9 (0.009)
SPD_0381 (acpP)	acyl carrier protein	ACP phosphopantetheine attachment site binding involved in fatty acid biosynthetic process		-1.9 (0.007)
SPD_0382 (fabK)	trans-2-enoyl-ACP reductase II	nitronate monooxygenase activity		-1.8 (0.01)
SPD_0383 (fabD)	malonyl CoA-acyl carrier protein transacylase	[acyl-carrier-protein] S-malonyltransferase activity		-2.2 (0.008)
SPD_0384 (fabG)	3-oxoacyl-(acyl-carrier-protein) reductase			-1.4 (0.05)
SPD_0387 (fabZ)	beta-hydroxyacyl-(acyl-carrier-protein)	3-hydroxyoctanoyl-[acyl-carrier-protein] dehydratase activity	fatty acid biosynthetic process	-1.6 (0.03)
SPD_0385 (fabF)	3-oxoacyl-[acyl-carrier-protein] synthase II	beta-ketoacyl-acyl-carrier-protein synthase II activity	fatty acid biosynthetic process	-1.9 (0.008)
SPD_0386 (accB)	acetyl-CoA carboxylase, biotin carboxyl carrier	acetyl-CoA carboxylase activity	fatty acid biosynthetic process	-1.5 (0.001)
SPD_0388 (accC)	acetyl-CoA carboxylase, biotin carboxylase	acetyl-CoA carboxylase activity/biotin carboxylase activity		-1.9 (0.05)
SPD_0389 (accD)	acetyl-CoA carboxylase, carboxyl transferase,	acetyl-CoA carboxylase activity/transferase activity	fatty acid biosynthetic process	-1.7 (0.05)
SPD_0390 (accA)	acetyl-CoA carboxylase, carboxyl transferase,	acetyl-CoA carboxylase activity/ATP binding	fatty acid biosynthetic process	-1.9 (0.03)
SPD_0856 (dgkA)	diacylglycerol kinase	diacylglycerol kinase activity	phospholipid biosynthetic process	-1.5 (0.05)
SPD_0347 (mvaD)	diphosphomevalonate decarboxylase	ATP binding/kinase activity	isopentenyl diphosphate biosynthetic process, mevalonate pathway	-1.7 (0.03)
**Protein folding**				
SPD_0459 (grpE)	heat shock protein GrpE	adenyl-nucleotide exchange factor activity	protein folding	-1.9 (0.02)
SPD_0460 (dnaK)	chaperone protein DnaK	ATP binding	protein folding	-2.1 (0.01)
SPD_1709 (groL)	chaperonin GroEL	ATP binding	protein refolding	-1.4 (0.03)
SPD_0461 (dnaJ)	chaperone protein DnaJ	ATP binding/zinc ion binding	DNA replication	-1.5 (0.05)
**de novo' pyrimidine nucleobase metabolic process**				
SPD_0608 (pyrF)	orotidine 5'-phosphate decarboxylase	orotidine-5'-phosphate decarboxylase activity	de novo' pyrimidine nucleobase biosynthetic process	-1.4 (0.05)
SPD_1133 (pyrB)	aspartate carbamoyltransferase	amino acid binding/aspartate carbamoyltransferase activity	de novo' pyrimidine nucleobase biosynthetic process	-1.4 (0.03)
SPD_0609 (pyrE)	orotate phosphoribosyltransferase	magnesium ion binding/orotate phosphoribosyltransferase activity	de novo' UMP biosynthetic process	-1.4 (0.01)
SPD_1548 (gmk)	guanylate kinase	ATP binding	purine nucleotide metabolic process	-1.5 (0.05)
SPD_0834 pyrH)	uridylate kinase	ATP binding/UMP kinase activity	de novo' CTP biosynthetic process	-1.4 (0.008)
**Thiamine biosynthetic process**				
SPD_0624 (thiE-1)	thiamine-phosphate pyrophosphorylase	magnesium ion binding/thiamine-phosphate diphosphorylase activity	thiamine biosynthetic process	-1.5 (0.007)
SPD_0628 (tenA)	transcriptional activator TenA, TENA/THI-4	thiaminase activity	thiamine metabolic process	-1.4 (0.01)
SPD_1779	thiamine pyrophosphokinase	thiamine binding	thiamine metabolic process	-1.5 (0.03)
**Cell division**				
SPD_0659 (ftsE)	cell division ATP-binding protein FtsE	ATPase activity/ATP binding	cell division	-1.7 (0.02)
SPD_1477 (ylmF)	YlmF protein	barrier septum assembly	barrier septum assembly	-1.5 (0.003)
SPD_1478 (ylmE)	YlmE protein			-1.4 (0.03)
SPD_1479 (ftsZ)	cell division protein FtsZ	GTPase activity	barrier septum assembly	-1.4 (0.05)
**Carbohydrate metabolism**				
SPD_0723 (rpiA)	ribose 5-phosphate isomerase A	ribose-5-phosphate isomerase activity	pentose-phosphate shunt, non-oxidative branch	-1.5 (0.03)
SPD_0790 (pyk)	pyruvate kinase	magnesium ion binding/potassium ion binding	glycolytic process	-1.4 (0.02)
SPD_1012 (eno)	phosphopyruvate hydratase	magnesium ion binding/phosphopyruvate hydratase activity	glycolytic process	-1.4 (0.01)
SPD_0420 (pflB)	formate acetyltransferase	formate C-acetyltransferase activity	carbohydrate metabolic process	-1.6 (0.03)
SPD_1823 (gap)	glyceraldehyde-3-phosphate dehydrogenase, type	oxidoreductase activity, acting on the aldehyde or oxo group of donors, NAD or NADP as acceptor	glucose metabolic process	-1.8 (0.04)
SPD_1582	sucrose-6-phosphate hydrolase, putative	sucrose alpha-glucosidase activity	carbohydrate metabolic process	-1.6 (0.006)
SPD_0143	UDP-glucose 6-dehydrogenase, putative	NAD binding/UDP-glucose 6-dehydrogenase activity	polysaccharide biosynthetic process	-2.1 (0.02)
SPD_0870	phosphoglycerate mutase family protein			-2.5 (0.009)
**Hydrolases**				
SPD_1076 (srtA)	sortase			-1.5 (0.005)
**Cellular amino acid biosynthesis**				
SPD_1209 (aroB)	3-dehydroquinate synthase	3-dehydroquinate synthase activity	aromatic amino acid family biosynthetic process	-1.6 (0.008)
SPD_1372	glyoxalase family protein			-1.4 (0.05)
SPD_0764 (sufS)	cysteine desulfurases, SufS subfamily protein	cysteine desulfurase activity/pyridoxal phosphate binding	cysteine metabolic process	-1.4 (0.009)
SPD_1899	glutamine amidotransferase, class 1	hydrolase activity	glutamine metabolic process	-1.5 (0.02)
**Catalytic activity**				
SPD_1411	isochorismatase family protein	catalytic activity		-1.4 (0.04)
SPD_1555	isochorismatase family protein	catalytic activity		-1.4 (0.01)
SPD_0852 (pyrDb)	dihydroorotate dehydrogenase, catalytic subunit			-1.4 (0.02)
**Homeostasis process**				
SPD_1714	thioredoxin family protein	protein disulfide oxidoreductase activity	cell redox homeostasis/glycerol ether metabolic process	-1.4 (0.05)
SPD_1464 (psaD)	thiol peroxidase	thioredoxin peroxidase activity	cell redox homeostasis	-2.2 (0.004)
SPD_1041 (nrdH)	glutaredoxin-like protein NrdH	electron carrier activity/protein disulfide oxidoreductase activity	cell redox homeostasis	-1.7 (0.03)
SPD_1028 (acoA)	TPP-dependent acetoin dehydrogenase	oxidoreductase activity, acting on the aldehyde or oxo group of donors, disulfide as acceptor		-1.4 (0.02)
SPD_0190 (nrdG)	anaerobic ribonucleoside-triphosphate reductase	[formate-C-acetyltransferase]-activating enzyme activity/4 iron, 4 sulfur cluster binding		-2.7 (0.02)
**Pathogenesis/cytolysis**				
SPD_1726 (ply)	pneumolysin	cholesterol binding	hemolysis of cells in other organism/pathogenesis	-1.5 (0.005)
SPD_1295	hemolysin			-2.7 (0.03)
SPD_0729	hemolysin-related protein	cholesterol binding	pathogenesis	-1.6 (0.005)
**Response to stress**				
SPD_1590	general stress protein 24, putative			-1.6 (0.001)
SPD_0667 (sodA)	superoxide dismutase, manganese-dependent	metal ion binding/superoxide dismutase activity		-1.4 (0.003)
SPD_1135 (nth)	endonuclease III	4 iron, 4 sulfur cluster binding/DNA-(apurinic or apyrimidinic site) lyase activity	base-excision repair	-2.1 (0.004)
**Cell wall organization and biogenesis**				
SPD_0853 (lytB)	endo-beta-N-acetylglucosaminidase precursor,	amidase activity/mannosyl-glycoprotein endo-beta-N-acetylglucosaminidase activity		-1.6 (0.05)
**Conserved hypothetical protein**				
SPD_0030	conserved hypothetical protein			-1.5 (0.001)
SPD_0145	conserved hypothetical protein			-1.4 (0.001)
SPD_0164	conserved hypothetical protein			-1.8 (0.01)
SPD_0181	conserved hypothetical protein			-1.4 (0.02)
SPD_0182	conserved hypothetical protein			-1.8 (0.009)
SPD_0256	conserved hypothetical protein			-1.6 (0.01)
SPD_0302	conserved hypothetical protein			-1.4 (0.002)
SPD_0339	conserved hypothetical protein			-1.5 (0.03)
SPD_0410	conserved hypothetical protein			-1.4 (0.05)
SPD_0425	conserved hypothetical protein			-1.9 (0.02)
SPD_0478	conserved hypothetical protein			-1.8 (0.05)
SPD_0488	conserved hypothetical protein			-2.2 (0.001)
SPD_0489	conserved hypothetical protein			-1.6 (0.007)
SPD_0499	conserved hypothetical protein			-1.4 (0.002)
SPD_0594	conserved hypothetical protein			-1.8 (0.008)
SPD_0681	conserved hypothetical protein			-1.3 (0.04)
SPD_0714	conserved hypothetical protein			-1.5 (0.03)
SPD_0791	conserved hypothetical protein			-2.1 (0.01)
SPD_0911	conserved hypothetical protein			-1.6 (0.05)
SPD_0929	conserved hypothetical protein			-1.7 (0.04)
SPD_0959	conserved hypothetical protein			-1.9 (0.02)
SPD_0962	conserved hypothetical protein			-1.4 (0.008)
SPD_0990	conserved hypothetical protein			-1.7 (0.02)
SPD_1003	conserved hypothetical protein			-1.4 (0.03)
SPD_1159	conserved hypothetical protein			-1.3 (0.05)
SPD_1171	conserved hypothetical protein			-1.4 (0.007)
SPD_1242	conserved hypothetical protein			-1.4 (0.04)
SPD_1294	conserved hypothetical protein			-2.8 (0.05)
SPD_1344	conserved hypothetical protein			-1.8 (0.03)
SPD_1380	conserved hypothetical protein			-1.2 (0.007)
SPD_1400	conserved hypothetical protein			-1.5 (0.02)
SPD_1558	conserved hypothetical protein			-1.6 (0.01)
SPD_1566	conserved hypothetical protein			-2.3 (0.01)
SPD_1588	conserved hypothetical protein			-1.6 (0.01)
SPD_1595	conserved hypothetical protein			-1.9 (0.004)
SPD_1662	conserved hypothetical protein			-1.6 (0.01)
SPD_1716	conserved hypothetical protein			-1.8 (0.008)
SPD_1718	conserved hypothetical protein			-2.7 (0.002)
SPD_1725	conserved hypothetical protein			-1.6 (0.004)
SPD_1728	conserved hypothetical protein			-1.8 (0.008)
SPD_1727	conserved hypothetical protein			-1.8 (0.005)
SPD_1729	conserved hypothetical protein			-1.6 (0.002)
SPD_1858	conserved hypothetical protein			-1.5 (0.007)
SPD_0855	conserved hypothetical protein			-1.4 (0.05)
SPD_1836	conserved hypothetical protein			-1.4 (0.05)

The functional annotation of the differentially regulated genes revealed that genes involved in galactose metabolism were exclusively up-regulated in pyrimidinedione-grown biofilms. Genes related to DNA replication, cell division and the cell cycle, pathogenesis, phosphate-specific transport, signal transduction, fatty acid biosynthesis, protein folding, homeostasis, competence, and biofilm formation were down-regulated. The fold change values of relative gene expression and predicted protein functions are detailed in Tables 2 and 3.

**Fig 10:**
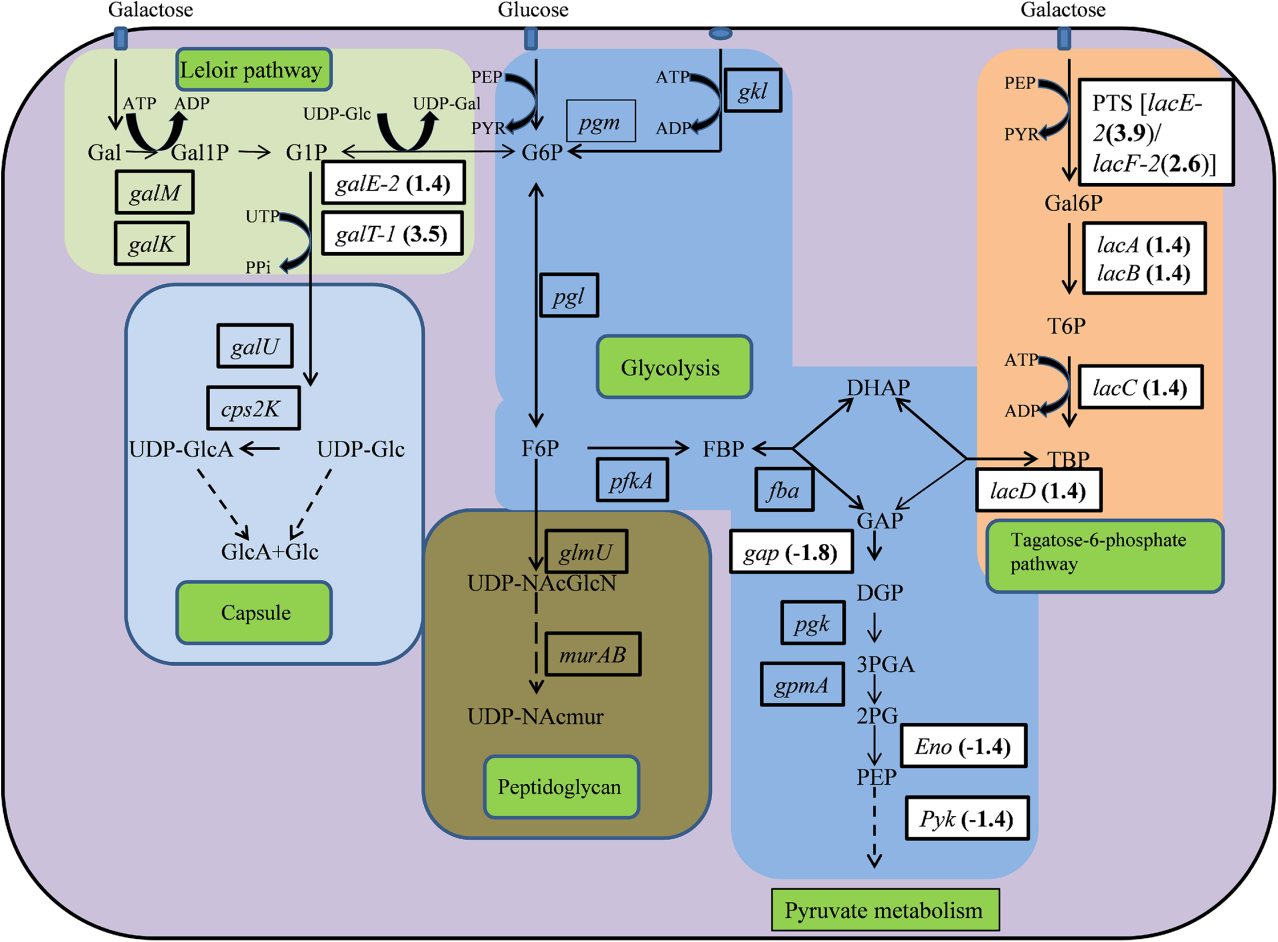
Schematic representation of central metabolic pathways in *Streptococcus pneumoniae* D39. In pneumococci, lactose and galactose are metabolized by the tagatose-6-phosphate pathway (light orange box) and the Leloir pathway (left; light green box). Tagatose-6-phosphate pathway genes include *lacA*, *lacB*, *lacC*, and *lacD*, and Leloir pathway genes include *galM*, *galK*, *galT-1*, and *galE-2*. Along with these the *lacF2* gene (encodes the PTS system, lactose-specific IIA component) and *lacE2* gene (encodes the PTS system, lactose-specific IIBC component) are required for galactose transport. The *gap* gene (encoding glyceraldehyde-3-phosphate dehydrogenase), *eno* gene (encoding phosphopyruvate hydratase), and *pyk* gene (encoding pyruvate kinase) are involved in glycolysis (central blue box). The *lacA*, *lacB*, *lacC*, *lacD*, *galT-1*, *galE-2*, *lacE-2*, *gap*, *eno* and *pyk* genes were down-regulated by more than 1.4 folds in this study. The relative fold-changes in gene expression are highlighted.

Nine genes involved in galactose metabolism were significantly up-regulated, while eight genes were down-regulated. Tagatose-6-phosphate pathway genes (*lacA*, *lacB*, *lacC*, *lacD*, and *lacG-2*) and Leloir pathway (*galT-1*and *galE-2*) genes were significantly up-regulated (Fig 10). In addition, the *lacF2* gene (encoding the PTS system, lactose-specific IIA component) and the *lacE2* gene (encoding the PTS system, lactose-specific IIBC components) were also up-regulated. However, the *gap* (encoding glyceraldehyde-3-phosphate dehydrogenase), *eno* (encoding phosphopyruvate hydratase), and *pyk* (encoding pyruvate kinase) genes involved in glycolysis were down-regulated.

**Fig 11:**
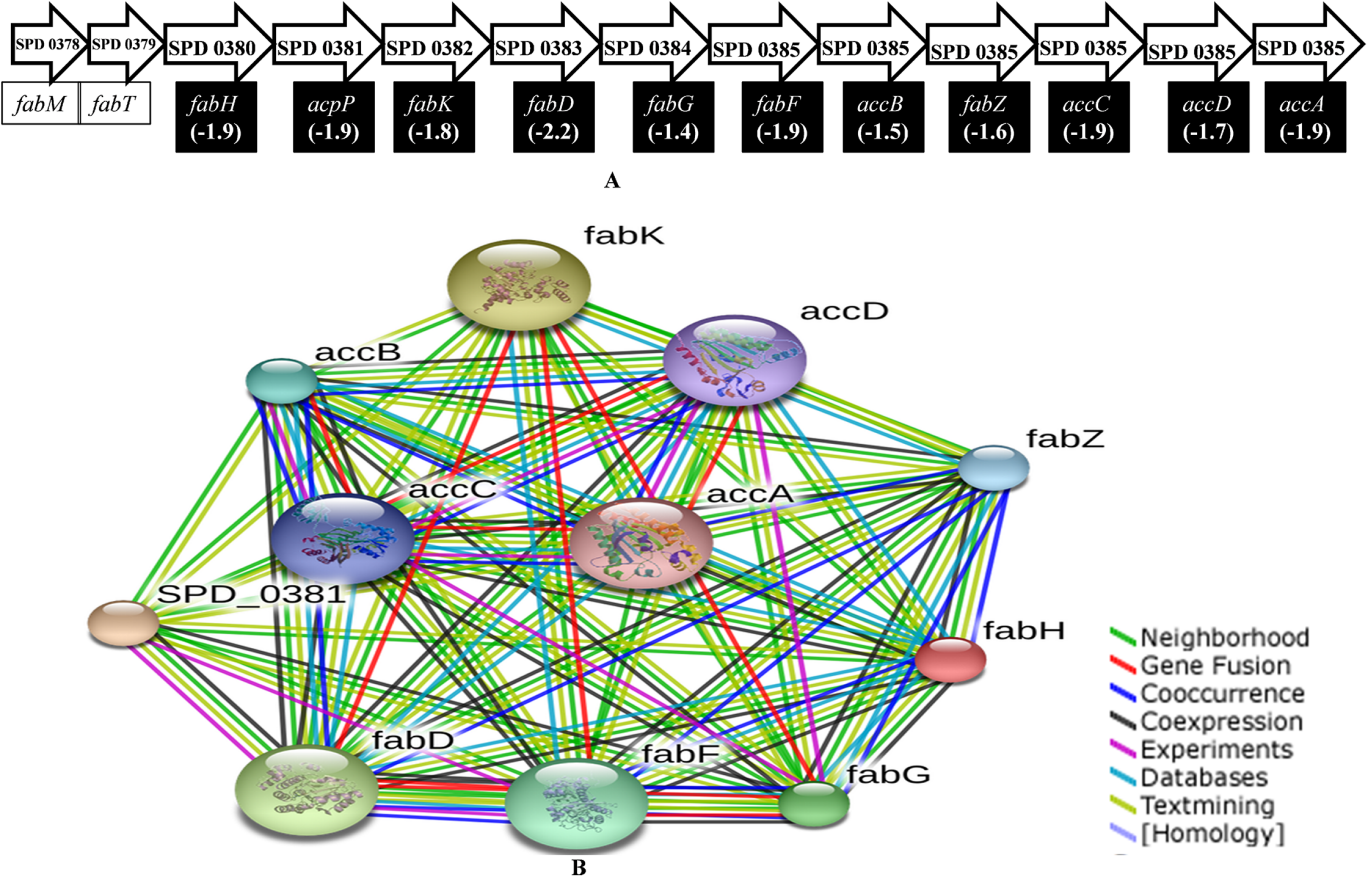
Fatty acid biosynthesis gene locus in *Streptococcus pneumoniae*. **(A)** FAS involves 13 genes that are arranged in a single locus that initiate fatty acid synthesis and product elongation. The fatty acid synthesis genes include: *acpP*, encoding acyl carrier protein (ACP); *accD*, encoding acetyl coenzyme A (acetyl-CoA) carboxylase subunit beta; *fabG*, encoding 3-ketoacyl-ACP reductase; *fabH*, encoding 3-oxoacyl-ACP synthase III; *fabK*, encoding trans-2-enoyl-ACP reductase II; *fabD*, encoding ACP S-malonyltransferase; *fabF*, encoding 3-oxoacyl-ACP synthase II; fabZ, encoding (3R)-hydroxymyristoyl-ACP dehydratase; accA, encoding acetyl coenzyme A (acetyl-CoA) carboxylase subunit alph; accB, encoding acetyl coenzyme A (acetyl-CoA) carboxylase subunit; and accC, encoding acetyl coenzyme A (acetyl-CoA) carboxylase subunit. (B) The interconnection of fatty acid genes detected by STRING v9.1 on the basis of terms on the right. The fold-changes in gene expression are highlighted.

The microarray results detected that expression of 11 genes belonging to the fatty acid synthesis (FAS) locus was significantly down-regulated (more than 1.4-fold) in pneumococcal biofilms grown in the presence of pyrimidinedione. No FAS pathway genes were up-regulated. The FAS pathway is made up of 13 genes arranged in a single locus that are involved in FAS initiation and product elongation (Fig 11).

Five genes involved in signal transduction were also down-regulated in pyrimidinedione-grown biofilms. Expression of the *ciaR* and *ciaH* gene, encoding a DNA-binding response regulator protein and a sensor histidine kinase respectively were down-regulated. These two genes encode the two-component regulatory system CiaH/CiaR, which is involved in the early steps of competence regulation. Expression of the *comC* gene, encoding the competence-stimulating peptide type 1, essential for pneumococcal competence and biofilm formation, was down-regulated 1.5-fold. Similarly, expressions of the *ptsH* gene, encoding the phosphocarrier protein HPr, and the SPD_0082 gene, encoding a sensor histidine kinase, were down-regulated 1.6- and 1.4-fold, respectively.

The expression of six virulence protein-encoding genes, *ply*, SPD_1295 (hemolysin), *nrc* (SPD_0729), *prtA*, *lytB*, and *srtA* was down-regulated in pyrimidinedione-grown biofilms. The expression of the *ply* gene was down-regulated 1.5-fold. The SPD_1295 and *nrc* genes were down-regulated by 2.7 and 1.6-fold respectively. Expression of the *prtA*, *lytB*, and *srtA* genes was down-regulated by 1.9, 1.6, and 1.5-fold respectively.

A large number of genes involved in transcription and DNA binding were down-regulated in pyrimidinedione-grown biofilms. Expression of 23 transcription and DNA binding genes was down-regulated, while five genes were up-regulated. Important down-regulated genes include, *ccpA*, *rpoC*, *comX2*, *bplS*, *spx*, and *cps2A*. The *ccpA* gene encodes catabolite control protein A, which is a negative repressor protein with a regulatory role in carbohydrate metabolism. The *comX2*gene encodes a sigma factor that functions as a competence-specific global transcription modulator involved in bacterial competence.

Other down-regulated genes encoded cell membrane proteins, as well as proteins involved in amino acid synthesis, catalytic activity, cell wall organization and biogenesis, homeostasis, response to stress, and thiamine and riboflavin biosynthesis.

### Quantification of gene expression by real-time RT-PCR

Thirteen differentially expressed genes from our microarray analysis were confirmed by real-time RT-PCR, and their differential expression levels were in agreement with the microarray data (Table 4).

**Table 4:** Gene expression analysis by real-time RT-PCR. Fold changes in gene expression of biofilms grown with pyrimidinedione with respect to control.

Gene	Mean fold change	p-value
*lacG-2*	1.5	0.05
*lacT*	3.5	0.05
*cglD*	2.2	0.05
*capD*	2.3	0.03
adk	2.5	0.03
*galT-1*	3.1	0.03
*purC*	2.2	0.02
*fabD*	-2.4	0.05
*dnak*	-2.0	0.04
*nrdG*	-3.0	0.01
*phoU*	-3.1	0.01
*pstB*	-1.8	0.03
*acpP*	-1.8	0.02

### Pyrimidinedione does not exhibit eukaryotic cell toxicity

**Fig 12:**
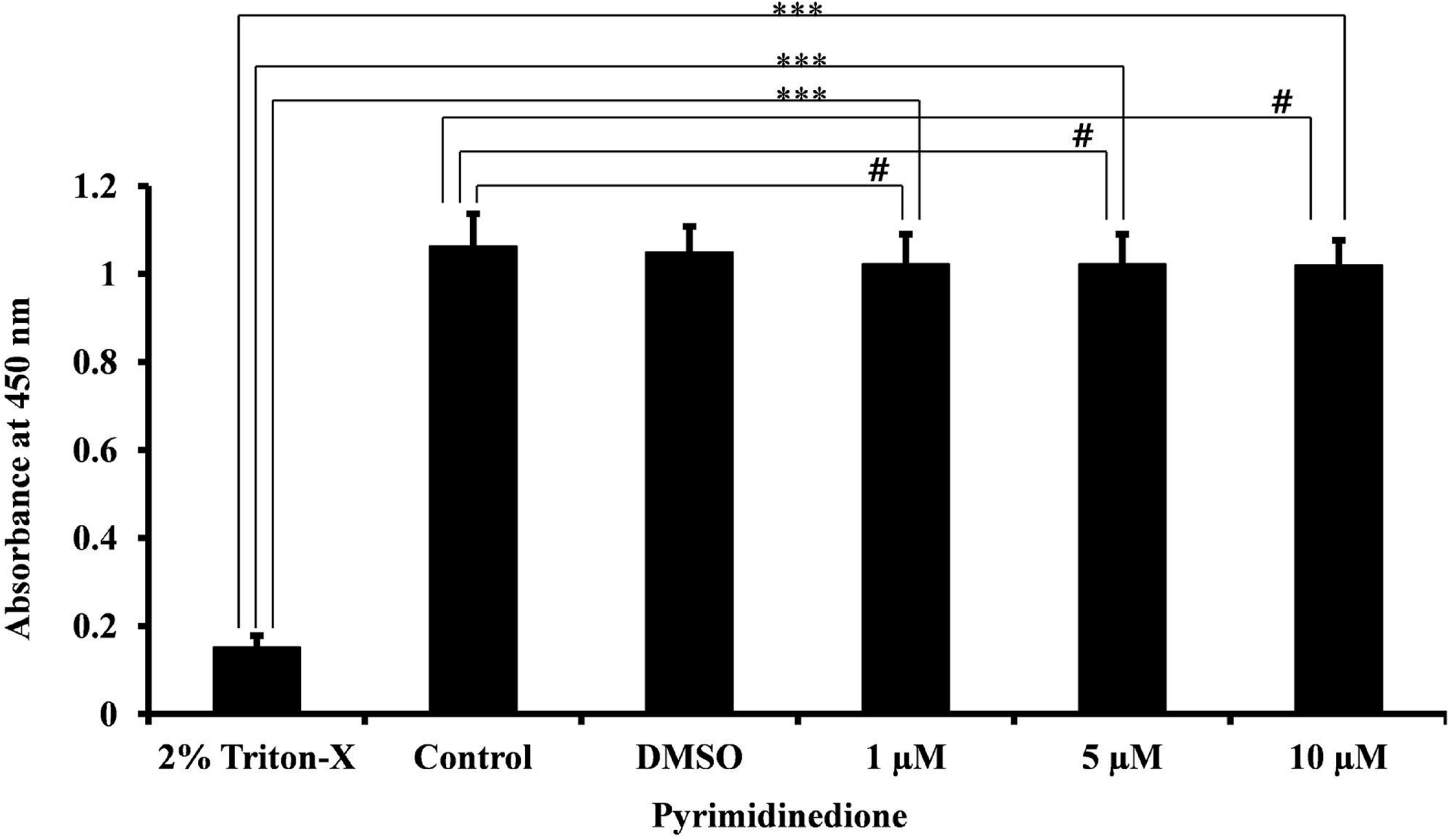
Cytotoxicity of pyrimidinedione on HMEECs. The cytotoxicity of pyrimidinedione was tested on the HMEEC line using a CCK-8 kit. The absorbance of the reaction was measured at 450 nm, and was compared among HMEECs exposed to pyrimidinedione (1 μM, or 5 μM, or 10 μM), 2% Triton X-100 (complete lysis), control (medium alone), and DMSO-control. No significant difference in mean absorbance was detected for HMEECs treated with various concentrations of pyrimidinedione versus untreated or DMSO controls (*p*> 0.43). However, all were significantly different from triton X-100-treated cells (*p*< 0.002), indicating no acute cellular cytotoxicity at the tested concentrations. The results were compared by Student’s *t*-test (*** corresponds to *p*< 0.002, ^#^corresponds to *p*>0.43). The error bars represent the SD.

The CCK8 cell viability experiment revealed no significant difference in mean absorbance of HMEECs treated with 1, 5, or 10 μM pyrimidinedione with respect to untreated or DMSO-controls (*p*>0.45). The absorbance of 2% triton X-100 treated HMEECs was significantly (*p*<0.002) lower than control- or pyrimidinedione-treated cells, indicating no acute cellular cytotoxicity at the tested concentrations (Fig 12). Thus, pyrimidinedione showed no evidence of acute toxicity to human HMEECs at a concentration of 10 μM, which was 10-fold greater than the established EC_50_.

## Discussion

*S*. *pneumoniae* are known to cause various biofilm-related infections in human. The physiology, metabolism, and gene expression profile of biofilm bacteria are different than planktonic bacteria (18). In *S*. *pneumoniae* biofilms, the quorum sensing (QS) signal generated by competence stimulating peptide (CSP) plays an important role in coordinating the spatial distribution of cells and the aggregation of exopolysaccharides [34,35]. Autoinducer-2 (AI-2) is the only QS molecule in pneumococci synthesized through activated methyl cycle (AMC), where Dam enzyme catalyze the transfer of a methyl group from SAM to macromolecules and adenine within DNA duplex [36,37,38]. Therefore, we hypothesized that interfering in Dam activity could have adverse effect on *S*. *pneumoniae* biofilms growth. Several studies reported that DNA adenine methylation regulates the expression of various virulence-related genes in numerous pathogens [39,40,41]. Our previous study demonstrated that interfering with methylation activity, either using the hypo-methylating agent (5-azacytidine) or a SAM analogue (sinefungin), inhibited pneumococcal biofilm growth. In this study, we examined the effect of the small molecule Dam inhibitor, pyrimidinedione, on *S*. *pneumoniae* biofilm growth. We then evaluated global gene expression changes within biofilms grown in the presence of pyrimidinedione.

Planktonic cell growth of *S*. *pneumoniae* D39 was not inhibited in the presence of different pyrimidinedione concentrations. The CV-microtiter plate assay and cfu counts detected a significant decrease in biofilm formation in samples treated with pyrimidinedione, and this inhibitory effect was concentration-dependent in all serotypes tested. The normal growth of planktonic cells and decreased biofilm formation in presence of pyrimidinedione indicated that pyrimidinedione selectively inhibits pneumococcal biofilms. Similarly, the Dam mutant strains of *Yersinia enterocolitica* and *Haemophilius influenza* showed reduced adhesion and host cell invasion capacity [42,43]. Pyrimidinedione was effective in inhibiting pneumococcal biofilm growth at both early and late stages [44]. Similar inhibitory effects of 5-aza-cytidine and sinefungin on *S*. *pneumoniae* biofilms as well as a small molecule adenosine mimetic on *Salmonella enteric* biofilms were previously reported [27,28,45]. However, pyrimidinedione was not effective in dismantling biofilms nor was it cytotoxic to bacteria within biofilms. *S*. *aureus* and *S*. *epidermidis* are important pathogens implicated in a wide variety of biofilm-related infections, including infections present within medical devices. The inhibitory effects of pyrimidinedione on *S*. *aureus* (MSSA and MRSA) and *S*. *epidermidis* biofilms indicated broad-spectrum anti-biofilm activity against antibiotic-resistant bacteria.

To understand biofilm changes at the microscopic level, we examined biofilms grown with and without pyrimidinedione by confocal microscopy and SEM. In microscopic analysis, the control and pyrimidinedione-biofilms demonstrated a significantly different morphology. The control biofilms were well organized and compact with significant thickness, and the cells were interconnected with each other and to the base of the plate [46]. A remarkable feature of these control biofilms was the presence of EPS [47]. EPS was completely absent in biofilms grown with pyrimidinedione. Due to lack of EPS, the cells were scattered, attached only to the bottom of the plate, and were unable to form an organized biofilm structure [48]. EPS is important for biofilm development; the absence of this structure indicated that bacteria were attached to the bottom of the plate. Therefore, it is possible that they could be easily washed away, decreasing biofilm biomass calculations and cfu counts [49].

In-order to examine the changes in gene expression of pyrimidinedione-grown biofilms, we evaluated global gene expression by microarray analysis. The overall gene expression pattern demonstrated that more genes were down-regulated in pyrimidinedione-grown biofilms compared to control biofilms. The gene expression analysis of 13 differentially expressed genes identified by microarray was confirmed using real-time RT-PCR. A functional annotation demonstrated that 17 functional gene groups were exclusively down-regulated, and four clusters were exclusively up-regulated in pyrimidinedione-grown biofilms.

The down-regulation of genes involved in DNA replication, cell division, cell organization and biogenesis, response to stress, homeostasis, and protein folding indicated that cell division may be perturbed and that cells were stressed in the presence of pyrimidinedione. In support of this hypothesis, 13 genes encoding ribosomal proteins showed reduced transcription in pyrimidinedione-grown biofilms, indicating that the pneumococci had reduced its translational capacity [50]. Similarly, bacterial transcriptional, signaling, and transport capacity may also be obstructed by down-regulation of transcription, DNA binding, transport, and signaling protein-encoding genes in pyrimidinedione-grown biofilms.

Fatty acid biosynthesis is essential for bacterial membrane integrity and cellular physiology, and the fatty acid biosynthesis gene mutant strains were unable to survive [51]. Pneumococcal fatty acid biosynthesis genes are collectively known as type II fatty acid synthase, which are clustered at a single location. The down-regulation of fatty acid biosynthesis genes in pyrimidinedione-grown biofilms indicated that disruption of fatty acid biosynthesis and product elongation may be affected.

A striking observation in our microarray and real-time RT-PCR gene expression results was the up-regulation of galactose metabolic pathway genes and down-regulation of glycolysis pathway genes. In pneumococci, lactose and galactose are metabolized by the tagatose-6-phosphate and Leloir pathways, respectively [52,53,54]. Our results demonstrated up-regulation of tagatose pathway genes, Leloir pathway genes, lactose-specific IIA & IIBC component encoding genes (*lacF2*and *lacE2*), and the *lacT* gene, encoding a transcriptional antiterminator. Conversely, we found down-regulation of glycolysis pathway genes in pyrimidinedione-grown biofilms. The up-regulation of galactose metabolism genes indicated that cellular carbohydrate metabolism was changed, and cells adapted to an alternative pathway. In pneumococci, the galactose and lactose metabolism pathway genes are up-regulated in the presence of sugars other than glucose [55]. The precise reason for the up-regulation of the galactose metabolic pathway genes in the presence of pyrimidinedione remains to be elucidated. However, it was reported that the transcription factor *CcpA* (carbon catabolite protein A), *ptsH*, encoding the phosphocarrier protein HPr, and the SPD_0082 gene, encoding a sensor histidine kinase, facilitates pneumococci utilization of diverse carbohydrate sources during colonization, multiplication, and biofilm formation [56,57]. Moreover, the regulation of the central carbohydrate metabolic pathway genes is under the control of carbon catabolite repression (CCR), which is mediated by the transcription factor CcpA and the histidine phosphoprotein HPr [58,59,60].

The down-regulation of five phosphate-specific transport system genes indicates that the transportation of molecules from the periplasm to the cytoplasm may be hindered by pyrimidinedione. Mutagenesis of the pst ABC genes in pneumococci resulted in decreased rates of phosphate uptake, decreased growth rates, decreased transformation, and reduced pathogenicity [61,62].

Here we detected the down regulation of virulence-related genes (*ply*, *srtA*, *ptrA*, *lytB*, *nrc*, and SPD_1295) in pyrimidinedione-grown biofilms. The *ply* gene is a virulence gene encoding the toxin pneumolysin, which causes eukaryotic cell lysis and plays a major role in pneumococcal invasion [63,64]. In *S*. *pneumonia*, the *srtA* gene is another virulence gene, and the *srtA* gene mutant strain showed low virulence and low adherence towards human pharyngeal cells [65]. The *lytB* gene encodes a choline-binding protein, and *nrc* and SPD_1295 encode hemolytic proteins. The *prtA* gene encodes the pneumococcal cell wall-associated serine protease A, which is important in virulence in intraperitoneal infections [66].

**Fig 13:**
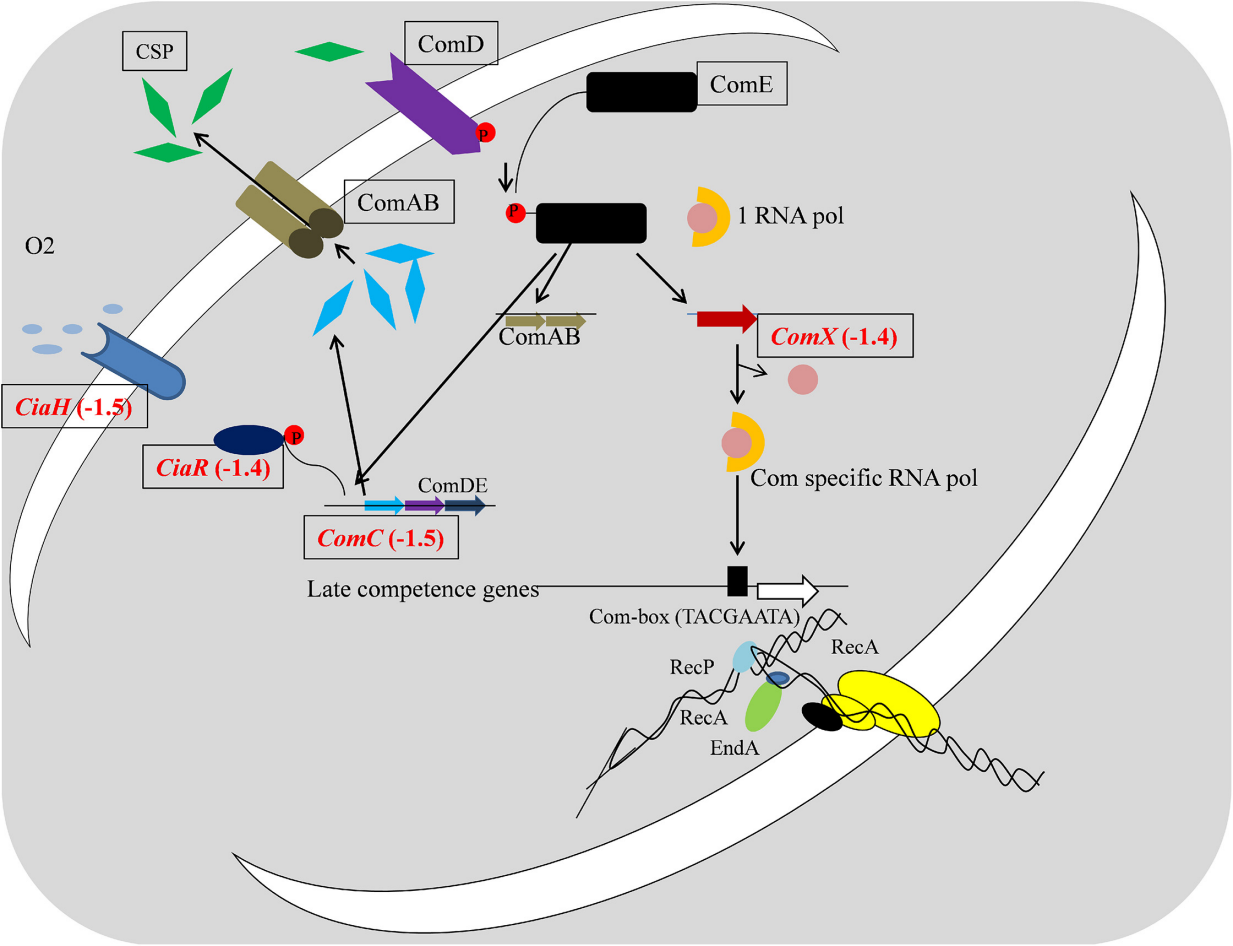
Schematic diagram representing genetic competence mediated by quorum sensing molecule competence specific peptide (CSP) in *S*. *pneumoniae*. Induction of genetic competence in pneumococci is regulated by a CSP–mediated quorum-sensing system. The precursor of CSP is encoded by the *comC* gene, and the ComAB (secretary and transporter) protein, facilities extracellular accumulation of mature CSP. Mature CSP then binds to ComD receptor, resulting in ComD auto-phosphorylation and phosphoryl group transfer to the response regulator, ComE. Phosphorylated ComE binds to the early gene promoter and activates the transcription of early genes. As a result of ComE binding, the transcription of the comCDE operon, and the production of CSP, ComD and phosphorylated ComE levels increase. ComE binding also initiates the accumulation of ComX (alternative sigma factor). ComX binds to the late gene promoter and stimulates the expression of late protein-encoding genes which facilitates recombination and DNA uptake. CiaH-CiaR is the second two-component regulatory system affecting the development of competence via regulation of *comCDE* expression. The fold-changes of gene expression are highlighted in red.

The significantly decreased biofilm growth observed in the presence of pyrimidinedione may be due to the decreased expression of competence and biofilm-related genes (Fig 13). Here, the competence stimulating peptide-1 (CSP-1) precursor encoding gene *comC*, the two-component regulator encoding genes *ciaH* and *ciaR*, and the alternative sigma factor encoding gene *comX* were down-regulated in response to pyrimidinedione treatment. In *S*. *pneumoniae*, the CSP-mediated QS system initiates the regulation of genetic competence, which involves the expression of early gene products encoded by *comAB* and *comCD* genes, and the two-component regulatory system CiaH-CiaR [67,68]. The pneumococcal *comC* gene encodes the CSP precursor and the *comDE* genes encode the CSP receptor and response regulator protein. The CiaH-CiaR negatively regulates *comCDE* expression and thus affects the development of competence. The response regulator comE binds to the early gene promoter and initiates transcription, as a results accumulation of CSP, ComD, phosphorylated ComE and ComX (a global transcription modulator) increases. The ComX alternative sigma factor initiates the transcription of late competence-specific operon, which facilities in DNA uptake and recombination of DNA [68,69]. Previous studies have reported that QS plays an important role in biofilm formation and a *ciaR/H* gene mutant strain was unable to form biofilms [49]. Oggioni *et al*. (2006) detected up-regulation of the *comC* gene in biofilms and demonstrated that *S*. *pneumoniae comC* mutants were less virulent and unable to form biofilms *in vitro*. They further reported that, when supplemented with external CSP-1, wild type levels of biofilm formation were restored in the mutant strain [18]. Similarly, in *S*. *mutans*, a *comC* mutant strain was unable to produce the signal peptide and biofilm formation was disrupted.

Our results indicate that the Dam inhibitor, pyrimidinedione, down-regulates the expression of various pathway genes including those involved in cellular metabolism, translation, transcription, cell division, amino-acid synthesis, virulence, and DNA replication. Previous studies report that disruption of Dam or Dam activity affects bacterial fitness and alters gene expression [39,40,41,70]. These perturbations were postulated to be indirect secondary effects of basic cellular fitness. As a result, pneumococcal planktonic cell growth was reduced but not completely inhibited. It is likely that the down-regulation of competence and biofilm-related genes resulted in lower levels of biofilm growth. These bacteria were unable to build organized biofilms or aggregate biofilm matrix [49].

## Conclusion

This study demonstrated that a small molecule Dam inhibitor, pyrimidinedione, perturbed pneumococcal biofilm growth *in vitro* at concentrations that did not inhibit planktonic cell growth and down-regulated the expression of important metabolic-, virulence-, competence-, and biofilm-related genes. Pyrimidinedione is also effective against MSSA, MRSA, and *Staphylococcus epidermidis* biofilm growth *in vitro*, and it is not cytotoxic to mammalian cells. Pyrimidinedione has potential for the development of new anti-biofilm compounds, and a ideal candidate molecule which require further study for *in vivo* biofilm prevention.
